# Endothelial TDP-43 depletion disrupts core blood–brain barrier pathways in neurodegeneration

**DOI:** 10.1038/s41593-025-01914-5

**Published:** 2025-03-14

**Authors:** Omar M. F. Omar, Amy L. Kimble, Ashok Cheemala, Jordan D. Tyburski, Swati Pandey, Qian Wu, Bo Reese, Evan R. Jellison, Bing Hao, Yunfeng Li, Riqiang Yan, Patrick A. Murphy

**Affiliations:** 1 Center for Vascular Biology, University of Connecticut Medical School, Farmington, CT USA; 2 Department of Pathology, University of Connecticut Medical School, Farmington, CT USA; 3 Center for Genome Innovation, University of Connecticut, Storrs, CT USA; 4 Department of Immunology, University of Connecticut Medical School, Farmington, CT USA; 5 Department of Molecular Biology and Biophysics, University of Connecticut Medical School, Farmington, CT USA; 6 Department of Neuroscience, University of Connecticut Medical School, Farmington, CT USA

**Keywords:** Mechanisms of disease, Neurodegenerative diseases, Transcriptomics, Cardiovascular biology

## Abstract

Endothelial cells (ECs) help maintain the blood–brain barrier but deteriorate in many neurodegenerative disorders. Here we show, using a specialized method to isolate EC and microglial nuclei from postmortem human cortex (92 donors, 50 male and 42 female, aged 20–98 years), that intranuclear cellular indexing of transcriptomes and epitopes enables simultaneous profiling of nuclear proteins and RNA transcripts at a single-nucleus resolution. We identify a disease-associated subset of capillary ECs in Alzheimer’s disease, amyotrophic lateral sclerosis and frontotemporal degeneration. These capillaries exhibit reduced nuclear β-catenin and β-catenin-downstream genes, along with elevated TNF/NF-κB markers. Notably, these transcriptional changes correlate with the loss of nuclear TDP-43, an RNA-binding protein also depleted in neuronal nuclei. TDP-43 disruption in human and mouse ECs replicates these alterations, suggesting that TDP-43 deficiency in ECs is an important factor contributing to blood–brain barrier breakdown in neurodegenerative diseases.

## Abstract (web summary)

Targeting the endothelium across ages and neurodegenerative diseases in 92 donors with single-cell inCITE-seq reveals similar alterations in ~40% of capillaries in AD, ALS and FTD. Endothelial TDP-43 loss coincides with increased nuclear p65/NF-κB activity and reduced Wnt/β-catenin.

## Main

The blood–brain barrier (BBB) tightly regulates the molecular and cellular composition of the central nervous system^1^. Endothelial cells (ECs) limit the passage of material with tight junctions and adaptor proteins, basal adherens junctions, tight regulation of general endocytic pathways as well as specific transporters and efflux pathways^1^. In aging, the BBB can become disrupted^2–4^ and in Alzheimer’s disease (AD), BBB dysfunction is more pronounced and accelerates more quickly^5^. Imaging studies show that BBB changes precede classical AD markers and brain atrophy^6–8^, suggesting that BBB disruption may be involved in AD onset. BBB dysfunction is not unique to AD and is observed in the pathogenesis of other neurodegenerative diseases, such as amyotrophic lateral sclerosis (ALS) and frontotemporal dementia (FTD)^9–11^.

ALS and FTD have an earlier onset than AD and are frequently driven by genetic lesions that directly or indirectly affect the nuclear function of the RNA-binding protein TAR DNA-binding protein 43 (TDP-43). The *TARDBP* gene product, TDP-43, is reduced in the neuronal nuclei in nearly all cases of ALS and approximately half the cases of FTD^12^. Many AD cases also exhibit a pattern of neuronal TDP-43 aggregation resembling FTD cases^13^. Notably in neurodegenerative disease, TDP-43 dysfunction is not confined to neurons, and is observed in astrocytes^14,15^, the pancreatic islet^16^ and even fibroblasts isolated from skin^17^. Whether nuclear levels of TDP-43 in ECs change with aging and neurodegenerative disease, and are linked to BBB disruption, has not been carefully examined.

Here, we target endothelial and microglial alterations in aging, ALS-FTD and AD, using a technique that we recently developed for the specific enrichment of endothelial nuclei from frozen brain tissues^18^. We combine this with intranuclear cellular indexing of transcriptomes and epitopes (inCITE-seq)^19^, to enable the measurement of nuclear proteins alongside single-nucleus RNA sequencing (RNA-seq).

## Results

### Erg-based enrichment of endothelial and microglial nuclei

**Fig. 1:**
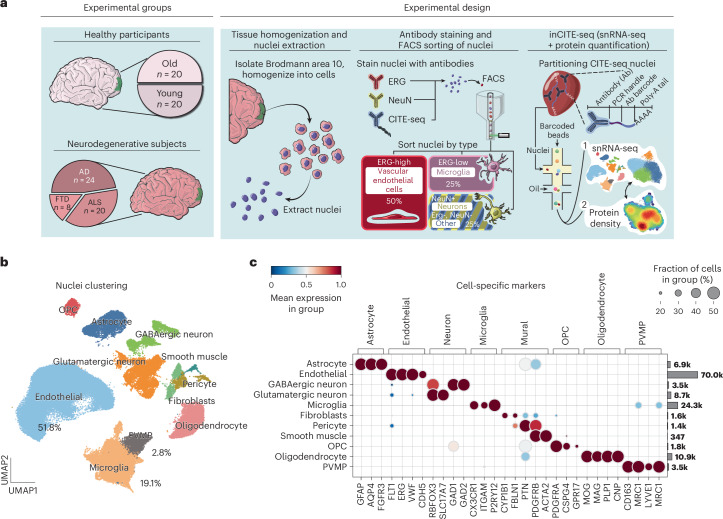
Single-nuclei analysis of human tissue samples with endothelial and microglial enrichment. **a**, Schematic representation of the methodology employed for endothelial enrichment, leveraging ERG and intranuclear CITE-seq antibodies. FACS, fluorescence-activated cell sorting. **b**, UMAP visualizing 132,859 nuclei from 92 human frontal cortex samples, color-coded by cell type. OPC, oligodendrocyte precursor cell; PVMP, perivascular macrophage. **c**, A dual representation featuring a dot plot of cell type-specific gene markers and a bar chart illustrating the count of captured nuclei for each cell type.

To enrich for ECs and microglia, which are typically underrepresented in single-nuclei analysis of the brain, we enriched them by staining for ERG transcription factor in an approach that we recently developed^18^. We obtained Brodmann Area 10 cortex samples from donors in the National Institutes of Health (NIH) NeuroBioBank, including young (*n* = 20, 15–29 years) and aged (*n* = 20, 67–95 years) donors without neurodegenerative diseases, or donors with neurodegenerative diseases (*n* = 24 AD, *n* = 20 ALS, *n* = 8 FTD; Supplementary Fig. 1 and Supplementary Table 1). Nuclei were stained per our protocol, but with modifications to allow for inCITE-seq labeling of nuclear proteins^19,20^ (see Methods and list of antibodies in Supplementary Table 2). Consistent with our previous work, we identified ERG^Hi^ and ERG^Lo^ nuclei, which did not vary significantly in abundance between conditions (Supplementary Fig. 2). ERG^Hi^ nuclei are endothelial and ERG^Lo^ nuclei, which likely represents ERG-like transcription factor FLI1 (ref. ^21^), are microglia^18^. We sorted the nuclei into four groups: endothelial (ERG^Hi^), microglial (ERG^Lo^), neuronal (NeuN)^22^ and other parenchymal nuclei (ERG^neg^NeuN^neg^). Then, these were recombined to achieve final proportions of 50% EC nuclei (~25× enrichment from pre-sort levels of 2% (ref. ^18^)), 25% microglial nuclei (~5× enrichment from pre-sort levels of 5% (ref. ^18^)) and a mixture of neuronal and other parenchymal nuclei (Fig. 1a). Nuclei were hashtagged in batches that included healthy young, aged and two types of dementia samples in each 10x reaction. After data processing, we observed proportions very similar to our expected nuclei input, with 51.8% endothelial nuclei and 22% microglial and macrophage nuclei and a median of 689 EC nuclei (Fig. 1b and Supplementary Figs. 3 and 4). The number of cells per donor and gene counts compared well with previous datasets^22,23^ (Supplementary Fig. 4). Cell types were delineated by known markers (for example ECs by FLT1 and ERG) (Fig. 1c). Addition of inCITE nuclear staining slightly reduced gene counts, but did not affect clustering of nuclei (Supplementary Fig. 5a,b). A more detailed analysis of the endothelial and mural compartments was able to subset these further into artery, capillary, vein and reactive capillary (Supplementary Fig. 6a) and pericytes, smooth muscle and either meningeal or perivascular fibroblasts (Supplementary Fig. 6b). Thus, ERG-based isolation provides a reliable method for the efficient interrogation of endothelial and microglial nuclei across a large set of brain samples.

### Clustering of sorted nuclei reveals disease-associated expression

**Fig. 2:**
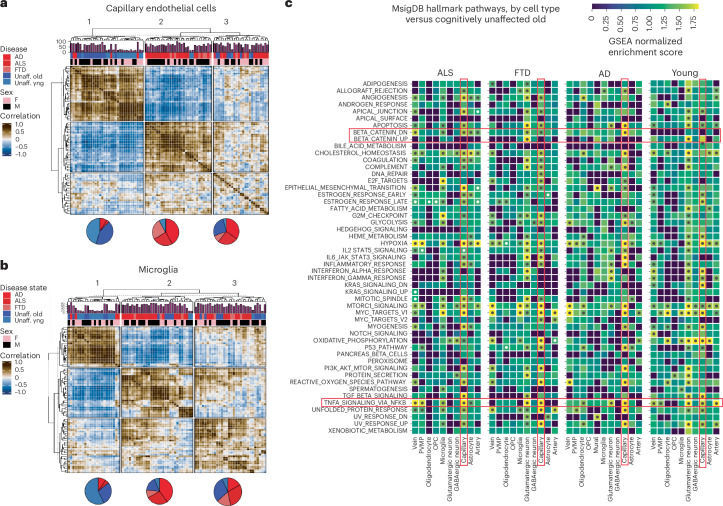
Principal-component analysis reveals shared transcriptional processes across neurodegeneration. **a**, Correlation matrix showing Pearson correlations of PCA-adjusted capillary EC profiles from 88 donors, having at least ten nuclei processed via Harmony for integration based on batches. The PCA, based on 50 components, is followed by hierarchical clustering and dendrogram visualization, categorizing data by sex and disease state. **b**, Similar correlation matrix for microglial population derived from 82 donors. Unaff., unaffected. **c**, Heatmaps showing top pathways enriched among the genes differentially expressed (pseudobulk) between each of the indicated donor populations compared to unaffected aged donors. Dots indicate differences in pathways and specific cell types where *P*adjusted < 0.05.

Microglial states correlate with the progression of AD, ALS and FTD^24–26^; however, less is known about the similarities or differences in EC transcriptional alterations across neurodegenerative diseases and aging. Existing datasets have examined the question, but typically with either a low number of EC nuclei across many samples^22^ or a large number of endothelial nuclei in a few samples^24^. Here, we leveraged the large number of samples across age and neurodegenerative diseases to determine the consistency in EC alterations in disease states and to compare these with healthy aging. We clustered capillary ECs from each donor by similarities in gene expression profiles using principal-component analysis (PCA) (Fig. 2a). This approach yielded three large clusters. The first cluster (1) was composed primarily of healthy donors. A second cluster (2) was highly enriched for clinically diagnosed dementia (AD and FTD). A third cluster (3) was composed of older controls, AD and ALS, with a low portion of FTD samples. This cluster seems to show a deterioration of the transcriptional pattern associated with cognitively normal donors, and a partial match to the pattern of expression observed in dementia samples. Major differences were independent of sex. Analysis of microglia (Fig. 2b), and other cell types, including venous ECs (Supplementary Fig. 7b) and astrocytes (Supplementary Fig. 7c), showed moderate disease-specific clustering, whereas arterial ECs (Supplementary Fig. 7a), oligodendrocytes (Supplementary Fig. 7d) and neurons (Supplementary Fig. 7e,f) exhibited much less.

To identify pathways implicated in neurodegeneration, we generated pseudobulk profiles for each donor by cell type, followed by DESeq2 analysis for differential expression (Supplementary Tables 3–6) and performed ranked gene set enrichment analysis (GSEA) on these profiles. We found that the capillary endothelium most frequently exhibited significant pathway alterations across disease states (Fig. 2c; dots indicate *P*adjusted < 0.05). For instance, β-catenin was notably downregulated in nearly all disease states, whereas tumor necrosis factor (TNF)/nuclear factor (NF)-κB signaling was increased (Fig. 2c). Other notable disease-associated alterations include increased transforming growth factor (TGF)-β, complement activation and misfolded protein response. Conversely, in aging, the glutamatergic neuronal population showed the most frequent pathway alterations followed by capillary and venule cell population. This further suggests that pathological transcriptional changes are more prominent in neurodegenerative conditions than in aging, particularly in capillary ECs (Fig. 2c). A comparison of differential gene expression in this data comparing AD to aged controls to previous datasets in human brain tissues revealed a significant overlap with data obtained from freshly isolated brain microvessels^23^ (>70% detection of these differentially expressed genes (DEGs) were also identified here; Supplementary Fig. 4d–f). Less overlap was observed with in silico-isolated brain EC compartments (<1% DGE; Supplementary Fig. 4d–f). Notably, the overall number of DEGs was expanded ~50-fold, likely reflecting increased donor numbers, cell numbers and transcript depth in the current data.

Thus, transcriptional profiles in both ECs and microglia correlate closely with disease progression in AD, ALS and FTD, but the transcriptional alterations in the capillary endothelium most reliably discriminate neurodegeneration from healthy aging.

### inCITE-seq detects high nuclear NF-κB in disease-associated microglia

**Fig. 3:**
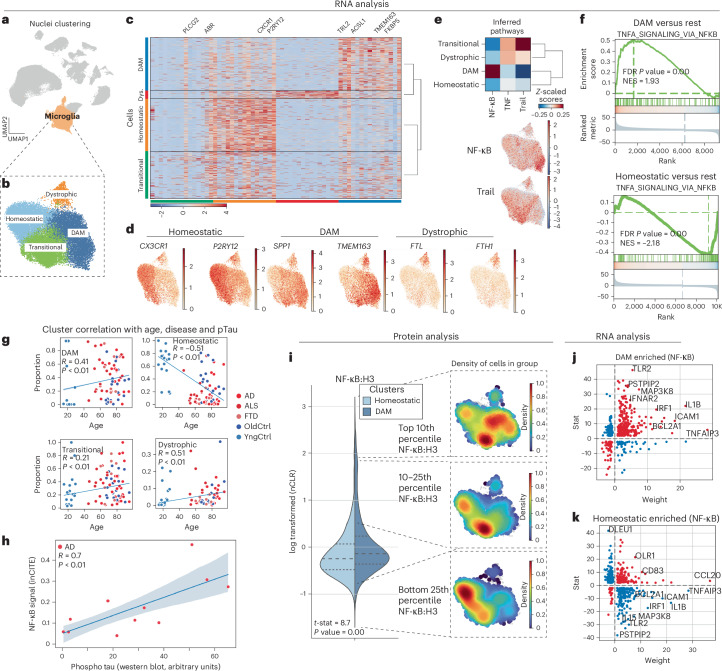
Microglial p65/NF-κB associates with disease-associated microglia. **a**, UMAP clustering representation of cortical microglial cells, derived from 22,588 cells from 90 donors (of which 9,479 cells from 45 donors belonging to inCITE dataset), with disease state regressed out. **b**, In silico isolation of microglia nuclei and UMAP clustering based on batch-corrected gene expression patterns, without regression of disease state. **c**, Heatmap illustrating differential gene expression patterns across the identified microglial subclusters. **d**, Gene counts for DAM, homeostatic and dystrophic microglial markers across cells. **e**, Comprehensive analysis of inferred pathways prevalent in the microglial clusters by PROGENY and mapping of inferred pathway scores onto cells in UMAP; red signal indicates higher expression of pathway genes. **f**, GSEA of the TNF signaling pathway within DAM and homeostatic clusters in contrast to other clusters from MSigDB. FDR, false discovery rate; NES, normalized enrichment score. **g**, Scatter-plot showing correlations between donor age (*x* axis), disease status (color) and their respective contributions to individual microglial clusters (*y* axis). **h**, Scatter-plot showing the correlation between NF-κB levels in the DAM cluster and pTau levels determined by western blot in the same brain tissue, with 95% confidence intervals (*P* = 0.014; *R* = 0.71). Two-sided Spearman correlation was used to assess the relationships. **i**, Violin plot showing the mean level of p65/NF-κB protein (normalized to levels of histone H3 protein) in cells of microglial DAM and homeostatic clusters and informatic isolation of nuclei with highest (top 10th percentile), high (top 10–25th percentile) and low (bottom 25th percentile) levels of NF-κB:H3 shown as a density plot. Areas of the UMAP enriched for cells falling into this range of NF-κB:H3 are red; lower levels are blue. **j**, Scatter-plot showing gene weight (relative association in the NF-κB pathway as predicted by the Progeny database, *x* axis) and log fold change between DAM cluster and other clusters (*y* axis). In red are transcripts increased or decreased in a manner consistent with NF-κB activation, and in blue are transcripts following a pattern of reduced NF-κB activity. **k**, Similar plot for homeostatic cluster. Stat, statistics.

Having identified disease-associated transcriptional changes in microglia, we aimed to assess the ability of the inCITE-seq approach to identify alterations in nuclear p65/NF-κB. Increased levels of microglial NF-κB signaling are observed in ALS, FTD and AD^27,28^. Extensive information is now available on microglial states observed by single-cell analysis in animal models and human tissues^26,29^; however, there is not yet data linking the microglial phenotypes to specific alterations in p65/NF-κB previously observed in human tissue samples^30^. To examine NF-κB protein levels in microglial subclusters, we computationally isolated microglia nuclei and then finely clustered them while regressing out batch effects. This resulted in four clusters of microglia, homeostatic, disease-associated microglia (DAM), transitional and dystrophic (Fig. 3a,b), as defined by characteristic gene expression^26,31^ (Fig. 3c,d). Homeostatic genes included *CX3CR1* and *P2RY12*; DAM-associated genes included *SPP1* and *TMEM163* along with downregulation of homeostatic genes and genes observed in dystrophic microglia phenotype, like *FTL* and *FTH*^32^. The transitional cluster is marked by genes from both homeostatic and DAM clusters. Analysis of inferred pathways implicated NF-κB, TNF and TRAIL among the top differential pathways, with NF-κB activity highest in the DAM cluster (Fig. 3e). Consistent with this, downstream canonical TNF/NF-κB transcriptional targets were elevated in the DAM cluster (Fig. 3f). Analysis of the proportion of each donor’s nuclei that contribute to each of the clusters revealed a significant enrichment of donors from AD, ALS and FTD in the DAM cluster (Fig. 3g). Thus, microglial clustering and specifically the enrichment of a DAM cluster in AD, ALS and FTD aligns well with expectations based on analysis of these cells in animal models.

We normalized antibody counts to histone H3 as a means of controlling for antibody access to the nuclear compartment. Nuclear pore proteins have been used for this purpose, but ALS samples exhibited a reduction in nuclear pore proteins, consistent with previous studies^33^, and levels of H3 were less variable (Supplementary Fig. 8). Nuclear protein levels are determined by both protein translation and stability and nuclear transport, and were generally poorly correlated with their mRNA transcripts (Supplementary Fig. 9), but correlated well with phospho-Tau (pTau) measured by western blot in brain tissue extract (Fig. 3h), consistent with a proposed positive interaction between pTau and NF-κB in microglia^27^. We predicted that nuclear levels would correlate with downstream transcriptional changes. Indeed, p5/NF-κB was increased in the DAM cluster relative to the homeostatic cluster (Fig. 3i). Moreover, we observed distinct enrichment of the top tenth percentile of p65/NF-κB in DAM and the bottom 25th percentile in the homeostatic clusters, consistent with the positive regulation of canonical TNF/NF-κB transcripts in DAM, and reduction in homeostatic clusters (Fig. 3e,f,j,k). Notably, we also detected a transitional cluster, marked by intermediate levels of p65/NF-κB and a TRAIL signaling response.

This validates the inCITE approach on the well-characterized microglial activation in AD, ALS and FTD, and extends previous work by showing that an NF-κB intermediate but TRAIL high transitional cluster exists in which the pathways activated are not canonical TNF/NF-κB response genes but more closely resemble the TRAIL signaling pathway.

### Transcriptional changes in neurodegenerative endothelium

**Fig. 4:**
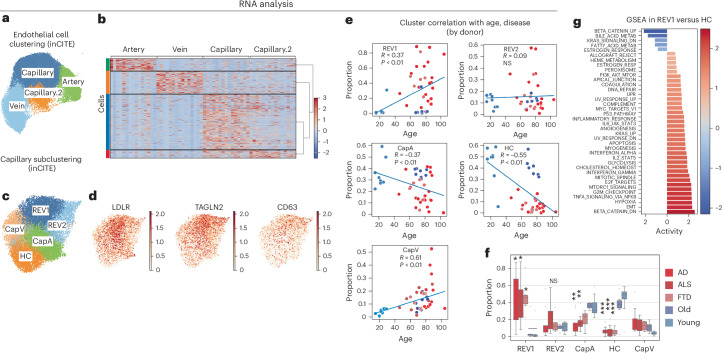
Distinct brain capillary endothelial states associate with healthy aging versus neurodegenerative diseases. **a**, UMAP clustering representation of brain EC subtypes, encompassing capillaries, veins and arteries, derived from 70,006 EC nuclei from 92 donors, 38,218 of these contain inCITE-seq antibodies, with disease state regressed out. **b**, Heatmap showing identification markers for capillary EC subtypes. Green, artery; orange, vein; blue, capillary; red, capillary.2. **c**, In silico isolation of capillary nuclei with inCITE antibody labeling and UMAP clustering based on batch-corrected gene expression patterns and without regression of disease state. Data represent 23,152 nuclei from 47 donors. **d**, UMAP feature plots showing markers of the REV1 population. **e**,**f**, Scatter-plot showing correlation (Spearman, two sided) between donor age and contribution to each capillary cluster (**e**) and box plot showing the proportions of capillary ECs for each donor falling into each cluster (**f**). **g**, Plot showing pathway enrichment from MSigDB in disease-associated REV1 cluster versus HC cluster, by GSEA. Color bar is normalized enrichment score. Analysis of variance (ANOVA) with a post hoc Tukey test was used to compare the proportion of donor’s cell per cluster (**f**). *P* values represent comparison to healthy aged donors. REV1 (AD *P* = 0.011; ALS *P* = 0.022; FTD *P* = 0.012). REV2 (NS). CapA (AD *P* = 0.002; ALS *P* = 0.008; FTD NS). HC (AD *P* = 0.0001; ALS *P* = 0.0001; FTD 0.0002). CapV (NS). ***P < 0.001; **P < 0.01; *P < 0.05; NS, not significant.

Alterations in ECs remain poorly annotated across age and disease states in the human brain, although animal models suggest a prominent role in both vascular aging and neurodegenerative disease^4,34–36^. However, our transcriptional data suggest that alterations within the endothelium are strongly correlated with disease states (Fig. 2). Therefore, we performed an analysis of brain ECs similar to the analysis we performed on microglia. ECs isolated by ERG^Hi^ included arteriolar, capillary and venular cells, marked by well-described markers (Fig. 4a,b and Supplementary Fig. 5a). The capillary cluster had the largest differences among disease states (Fig. 2a,c). Similar to our approach with microglia, we subclustered the capillary ECs and identified five distinct major clusters (Fig. 4c and Supplementary Fig. 10a,b). The identities of these capillary subclusters were determined by examining the top genes expressed in each cluster (Supplementary Fig. 10b), and were identified as arteriolar (CapA, for example *ALPL*, *ARL15* and *VEGFC*), venular (CapV, for example *PDE7B* and *NPIPB5*) and reactive (REV1 and REV2, for example *IFITM3*, *IRF1*, *TNFRSF6B* and *TMSB10*) or homeostatic (HC, for example *HMCN1*, *CCDC141* and *PROM1*). Reactive and homeostatic clusters were defined by enrichment of neurodegenerative or healthy donors respectively (Fig. 4e,f). Reactive post-capillary venules (REV) were recently defined in mice^37^. We observed LDLR among the top REV1 genes; LDLR has high affinity to ApoE protein and regulates in amyloid levels in the brain^38^. This coincided with the expression of TAGLN2 and CD63, which are involved in cytoskeletal organization following EC activation^39,40^ (Fig. 4d). The age of the donor had little effect on the contribution of cells to these clusters, as both young donors and cognitively normal aged donors exhibited a heavy skewing toward the HC cluster and away from REV1. Together, these two clusters represent 47% of all of the capillary ECs from each donor (Fig. 4f) and are therefore a large portion of the capillary bed. REV1 associated pathways include epithelial-mesenchymal transition, G2M checkpoint, TNF/NF-κB signaling (Fig. 4g) and reduced Wnt/β-catenin. In comparison, the HC cluster is characterized by genes associated with increased β-catenin signaling and lipid metabolism. Thus, analysis of capillary EC populations reveals a switch to proinflammatory capillary subtype in neurodegenerative diseases. This REV1 cell cluster consists of capillary ECs with transcriptional profile indicative of BBB disfunction.

### Neurodegenerative endothelial cells exhibit reduced Wnt/β-catenin and TDP-43

**Fig. 5:**
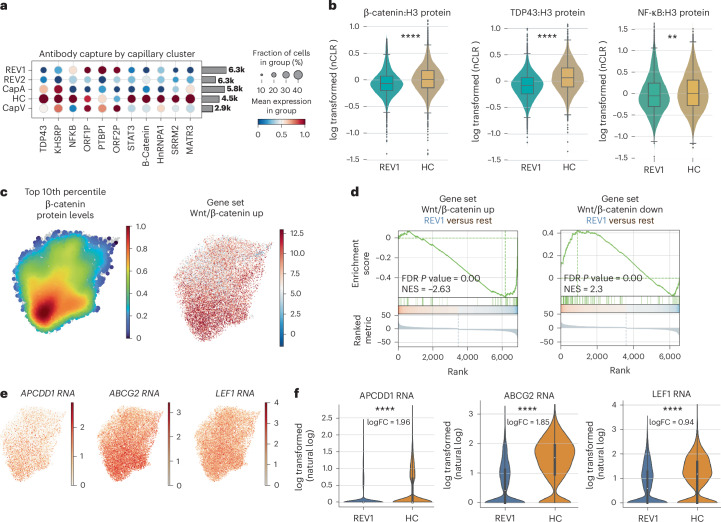
inCITE analysis reveals a loss of Wnt/β-catenin signaling in disease-associated nuclei. **a**, Dot plots showing relative levels of protein across all cell clusters and within the endothelial and microglial clusters. **b**, Violin plot showing histone-normalized protein levels for β-catenin, TDP-43 and p65/NF-κB in REV1 cluster compared to HC. REV1 cluster represents 6,297 nuclei and HC cluster represents 4,507. The distribution of those protein levels displayed in box plot shows interquartile range (IQR) (25th–75th percentiles) with the median (50th percentile) as the center line, whiskers extending to the minimum and maximum values within 1.5 × IQR, and individual points representing outliers beyond these bounds. Unpaired *t*-test used to evaluate significance. β-catenin (*t* = 12.91, *P* = 7.36 × 10^−38^, Cohen’s *d* of 0.252); TDP-43 (*t* = 31.96, *P* < 2.71 × 10^−214^, Cohen’s *d* of 0.624); p65/NF-κB (*t* = 4.43, *P* < 9.10 × 10^−06^, Cohen’s *d* of 0.087). **c**, Density plots illustrating the top 10th percentile of protein levels for β-catenin (relative to H3), and predicted positive regulation of Wnt signaling pathway by gene expression. **d**, GSEA plot of Wnt/β-catenin genes enriched in disease-associated REV1 cluster (versus all other clusters). Gene sets are either genes positively associated with Wnt activation in human ECs or negatively associated with Wnt activation. **e**, Feature plots showing example genes in the β-catenin pathway, projected onto the capillary UMAP. **f**, Violin plots with inner box-and-whisker plot with default parameters showing Wnt target gene expression in REV1 and HC clusters. REV1 cluster represents 6,297 nuclei and HC cluster represents 4,507. The rank_genes_groups function was used to derive statistics between cluster REV1 and HC using a *t*-test. APCDD1 (logFC = 1.96, *P*adj = 4.14 × 10^−139^), ABCG2 (logFC = 1.85, *P*adj = 0.00), LEF1 (logFC = 0.94, *P*adj = 1.58 × 10^−135^). FC, fold change.

The inclusion of inCITE-seq antibodies allowed us to take this analysis one step further (Fig. 5a), and to assess key markers of endothelial activation (p65/NF-κB), barrier function (β-catenin/Wnt) and TDP-43 across these clusters. Quantification of antibody staining intensity in the capillary cluster showed an increase in nuclear β-catenin protein level in the HC cluster and a decrease in REV1 cluster (Cohen’s *d* of 0.252 HC versus REV1), a trend similar to TDP-43 (Fig. 5a,b; Cohen’s *d* of 0.624 HC versus REV1). Uniform Manifold Approximation and Projection (UMAP) representation, which shows the density of cells within the top tenth percentile of β-catenin, suggested a clear increase in nuclear β-catenin in HC cluster compared to the rest (Fig. 5c). Nuclear levels of β-catenin coincided with an enrichment of upregulated transcriptional targets of β-catenin in the HC cluster (Fig. 5c,d). For example, key transcriptional targets of β-catenin signaling like TCF/LEF1, ABCG2 and APCDD1 were significantly higher in HC cluster than REV1 (Fig. 5e,f). Thus, the REV1 population, which is highly enriched for AD, ALS and FTD donors, exhibits reduced levels of TDP-43 and nuclear β-catenin, as well as reduced expression of canonical Wnt response genes.

### Impaired coordination of p65/NF-κB and TDP-43 in neurodegeneration

There was a small but statistically significant increase in p65/NF-κB in the HC cluster relative to REV1 (Fig. 5b; Cohen’s *d* of 0.087); however, there was a prominent and opposite increase in NF-κB transcriptional targets in the REV1 cluster versus the HC cluster (Fig. 4g). To shed light on this paradox, we obtained insight from our parallel work and another study of TDP-43 function in animal models and cultured cells^41,42^. These studies indicated that a reduction in TDP-43 leads to increased activation of p65/NF-κB transcriptional targets (and decreased β-catenin signaling) in brain ECs at steady state. Thus, the reduction in TDP-43 we had observed in the nuclei in the REV1 cluster might regulate the transcriptional response associated with nuclear p65/NF-κB. Therefore, we examined the level of nuclear TDP-43 together with p65/NF-κB by our inCITE-seq approach.

As TDP-43 lacks the well-defined transcriptional output of NF-κB and Wnt, we asked whether TDP-43-bound transcripts were enriched among those correlated with TDP-43 expression. We found that TDP-43 CLIP targets were enriched in transcripts positively correlated with TDP-43 (Supplementary Fig. 11a; odds ratio 4.64, Fisher’s exact test, *P* < 0.0001). This is consistent with the known effect of TDP-43 on the suppression of cryptic exons in genes, which are often mis-spliced and targeted for nonsense-mediated decay upon TDP-43 loss. Among those genes are *SORT1*, which was shown to be mis-spliced in cases of TDP43 dysfunction^43^. In addition, transferrin receptor (*TFRC*) and lipid transporter (*ATP10A*) are among the top genes showing positive association with TDP-43 protein levels, suggesting that the production of functional transcripts from these genes may also be regulated by TDP-43. We also directly validated TDP-43 specificity by applying our inCITE-seq antibody panel to human brain ECs (HBEC5i) with siRNA-mediated suppression of TDP-43, and found a reduced signal (Supplementary Fig. 12a). Furthermore, as nuclear levels of TDP-43 are reduced in >90% of cases of ALS and >50% of AD, we examined nuclear levels of TDP-43 in neuronal nuclei. We found a reduction in ALS and AD nuclei as well (Supplementary Fig. 12b). Thus, our inCITE-seq measurement of TDP-43 accurately reflects experimental and expected loss of nuclear TDP-43 and effects on known TDP-43-bound transcripts.

**Fig. 6:**
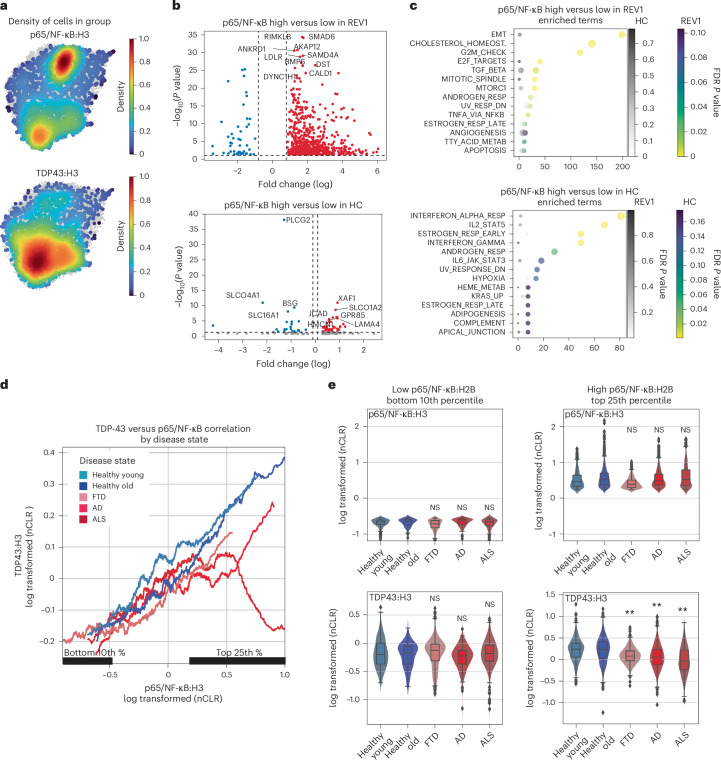
inCITE-seq analysis reveals a specific loss of nuclear TDP-43 and increased NF-κB transcriptional targets in disease-associated nuclei. **a**, Density plots illustrating the top 10th percentiles of protein levels for p65/NF-κB and TDP-43. **b**, Transcripts positively associated with nuclear p65/NF-κB in each cluster. *P* values reported on the *y* axis are derived from a *t*-test on differential gene expression between the two groups. **c**, Pathway enrichment, by Enrichr, shows MSigDB pathways enriched in nuclei in top 10th percentile of nuclear p65/NF-κB compared to the bottom in REV1 and HC clusters. Enrichment of the pathways shown for each cluster is also shown in gray for the other cluster. *P* values (FDR *q*-values) are indicated as color scales, and derived from hypergeometric analysis using Enrichr. **d**, A smoothed line plot comparing NF-κB protein levels (*x* axis) to TDP-43 (*y* axis). **e**, Violin plot showing histone-normalized protein levels for p65/NF-κB and TDP-43 in the bottom tenth percentile (2,208 nuclei) and top 25th percentile (5,780 nuclei) of p65/NF-κB levels within the capillary endothelial cell cluster. Box plots display the IQR (25th–75th percentiles) with the median (50th percentile) as the center line and whiskers extending to the minimum and maximum values within 1.5 × IQR. ANOVA with Tukey’s HSD post hoc tests was used to assess significance. Significance of TDP-43 protein levels in the bottom tenth percentile (bottom left) and top 25th percentile (bottom right) of NF-κB/p65 nuclear protein in capillaries relative to healthy old donors is shown. TDP-43 levels showed a significant difference between aged and young donors (*P* < 0.001) (bottom left). TDP-43 levels were significantly different in AD (*P* < 0.001), ALS (*P* < 0.001) and FTD (*P* < 0.001), but not in young controls (*P* > 0.05) (bottom right).

We then examined how the levels of nuclear TDP-43 changed across capillary clusters. In particular, we focused on the cells with the highest level of nuclear p65/NF-κB, which we believed would be driving the transcriptional NF-κB response detected in our data. We found that although there were high levels of nuclear p65/NF-κB in both REV1 and HC clusters, this was associated with a reduction in TDP-43 specifically in the AD, ALS and FTD-enriched REV cluster1 (Fig. 6a). We also noted a different distribution in the density plot, with p65/NF-κB high cells in disease REV1 being tightly packed, but distributed in HC cluster. As UMAP clustering proximity reflects similarities in gene expression patterns in each nucleus, this suggests a strong and conserved transcriptional signature across cells with high levels of nuclear p65/NF-κB in diseased ECs in REV1, but not in the HC cluster. In line with this observation, we observed strong alterations in gene expression with increasing levels of p65/NF-κB in REV1, but not the healthy HC cluster (Fig. 6b). Notably, the specific transcripts and pathways were also different, and pathways associated with p65/NF-κB levels in REV1 diseased ECs included EndoMT, TGF-β and canonical TNF/NF-κB signaling, while the pathways associated with NF-κB in healthy ECs of the HC cluster included interferon, JAK/STAT and apical junction (Fig. 6c).

To examine the relationship between TDP-43 and NF-κB more closely by disease state, we plotted the relationship of NF-κB to TDP-43 levels in each sample type, hypothesizing that a stoichiometry may need to be maintained between their nuclear levels. On these plots, a continuum of cells can be observed with a proportional increase in TDP-43 with NF-κB (Fig. 6d). This relationship was almost identical in cognitively normal donors, young or old, and AD, ALS and FTD donors, until NF-κB levels rise to the highest levels (top 25th percentile, Fig. 6d). At this point, the relationship broke down, most prominently in ALS and to a lesser extent in AD and FTD, and the levels of TDP-43 relative to NF-κB dropped (Fig. 6d, with quantitation in Fig. 6e). Within the vascular compartment, this relationship was strongest in capillaries, relative to arteries and veins (Supplementary Fig. 13a,b). Notably, these subsets included a large number of nuclei from several donors, indicating similar responses across the disease group (Supplementary Fig. 14).

Together, our data show that nuclear levels of TDP-43 in capillary ECs are reduced specifically in AD, ALS and FTD donors at the highest levels of NF-κB, and that this correlates with an increased level of TNF/NF-κB target genes in these cells.

### Targeted depletion of TDP-43 mimics disease-associated changes

**Fig. 7:**
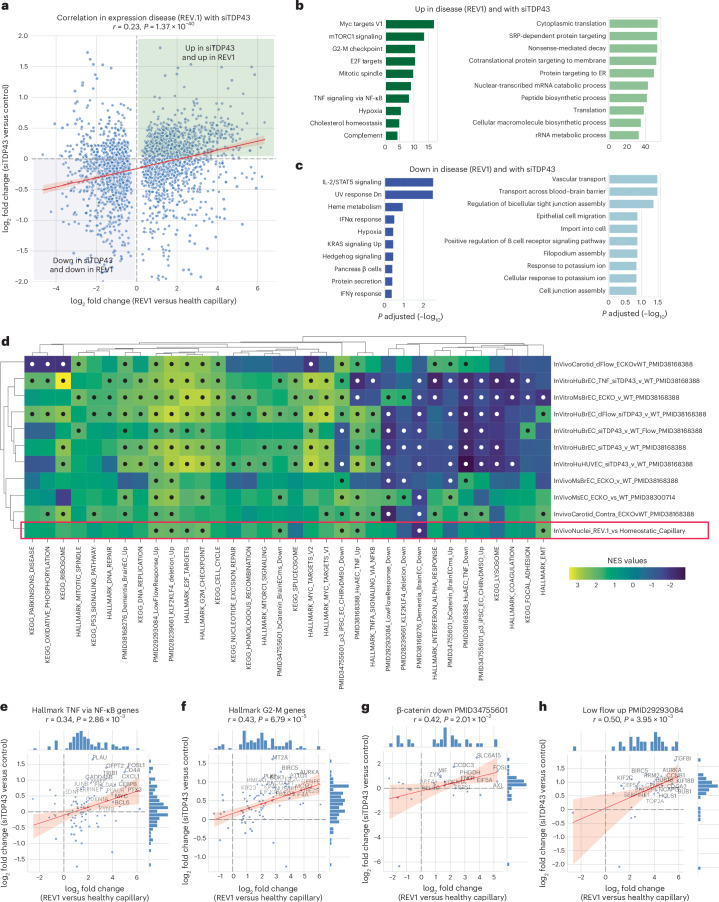
Correlation of transcriptional effects of TDP-43 loss with dementia capillary signature. **a**, Correlation between the effect of TDP-43 depletion on mRNA transcript level in human brain ECs (*y* axis) and the same mRNA affected in the comparison of REV1 and HC endothelial clusters (*x* axis). Human brain ECs were treated with no siRNA, nontargeting siRNA and two different TDP-43 siRNA across four biological perturbations (static culture, TNF stimulation and laminar or disturbed flow; *n* = 4 per treatment, *n* = 8 without TDP-43 knockdown and *n* = 8 with knockdown). **b**,**c**, Enriched Hallmark (dark green and dark blue) and Gene Ontology (GO) terms (light green and light blue) for transcripts increased in REV cluster and by siTDP-43 (**b**) or decreased in REV cluster and by siTDP-43 (**c**). Enrichr in GSEApy was used to assess values from hypergeometric test of overlap between regulated transcripts and the indicated msigDB databases. **d**, Heatmap showing the results of GSEA of Hallmark and Kegg pathways, and a set of custom EC response profiles (for example, disturbed flow and β-catenin activation) from the literature. NES derived from ranked and weighted (=1) GSEA analysis of all expressed transcripts (DESeq2 base mean 200). DESeq2 ranked lists and GSEA sets (KEGG, HALLMARK and custom) are reported in the supplementary tables. Dots indicate FDR *q*-value significance from GSEA. **e**–**h**, Focused plots showing the correlated response in specific pathways, between disturbed flow ECs with and without siTDP-43. Pathway genes shown are Hallmank TNF via NF-κB (**e**), Hallmark G2-M transition (**f**), top transcripts suppressed by activation of Wnt/β-catenin in PMID34755601 (**g**) and top transcripts induced by exposure of carotid endothelium to low and disturbed flow in PMID29293084 (**h**). Scatter-plots show data points and trend line with 95% confidence intervals (**a**,**e–h**). Histograms (**e**–**h**) show the fraction of points within the indicated interval on each axis. Two-sided Pearson correlations and *P* values are shown.

Our data from human samples, while correlational, suggest that a loss of nuclear TDP-43 in capillary ECs may alter the cellular response to nuclear p65/NF-κB levels, and could also be linked to changes in Wnt/β-catenin signaling; however, isolating alterations in TDP-43 from the other changes in AD, ALS and FTD is challenging, making causal attribution unclear. Therefore, we aimed to directly compare the effects of the loss of TDP-43 to transcriptional changes occurring in REV1 capillary cluster with reduced TDP43. We used data obtained from hTert immortalized human brain ECs (Supplementary Table 7), with siRNA-mediated suppression of TDP-43 (*n* = 8 versus *n* = 8) and mice with the deletion of TDP-43 (*n* = 6 versus *n* = 6, and *n* = 3 versus *n* = 3)^43,44^. For these data, we performed differential gene expression by DESeq2 to obtain significance and log_2_ fold change. Overall, there was a moderate correlation between transcriptional changes induced by the loss of TDP-43 and those observed in REV1 nuclei (Fig. 7a; *r* = 0.23, *P* = 1.37 × 10^−40^). Analysis of the pathways most enriched in REV1 and with experimental depletion of TDP-43 by Enrichr analysis included the Myc pathway, mesenchymal transition and TNF/NF-κB signaling (Fig. 7b). The most depleted pathways included interleukin (IL)-2–STAT5, regulation of tight junctions, and transport across the BBB (Fig. 7c). A ranked gene assessment of altered transcriptional pathways between conditions with and without genetic or siRNA-mediated TDP-43 disruption (Supplementary Table 7) and REV1 revealed consistent effects in several pathways, but most notably the shear-stress response mediated by KLF2/KLF4, TNF/NF-κB signaling and CHIR/Wnt agonist (Fig. 7d and Supplementary Fig. 15).

We hypothesized that while overall gene expression patterns may exhibit some similarities between REV1-associated transcriptional changes and the loss of TDP-43 (as in Fig. 7a), transcripts associated with pathways driving the larger transcriptional changes may show an even tighter overlap. To examine this, we isolated genes associated with individual pathways, and re-plotted the correlation between individual datasets (Fig. 7e–h and Supplementary Fig. 16). We found that in many cases, the correlation improved beyond the overall transcriptional correlation (for example *r* = 0.43 for Hallmark G2-M genes in Fig. 7f, versus *r* = 0.23 in Fig. 7a). This approach also highlighted a general increase, in both datasets, of the same set of transcripts.

Thus, pathway analysis of transcriptional changes associated with the loss of TDP-43 across a large set of biological contexts, in vitro and in vivo, and in human and murine cells, indicates conserved effects on the same pathways we found were altered in REV1. These data imply that the loss of TDP-43 observed in REV1 is sufficient to drive most changes in key pathways, including Wnt/β-catenin and TNF/NF-κB, which were observed in these dementia-associated nuclei. This analysis further nominates an impairment in flow-mediated signaling as a key component of the observed endothelial dysfunction.

## Discussion

Here, we show that nearly half of the capillary ECs across a range of neurodegenerative disease states, including AD, ALS and FTD, exhibit a unique transcriptional signature. By applying inCITE-seq analysis of TDP-43 and key transcription factors, we reveal that an increased canonical NF-κB transcriptional output in disease-associated capillary ECs is not associated with increased levels of nuclear NF-κB, but is instead associated with a drop in nuclear levels of TDP-43 at the highest levels of NF-κB. This correlates with increased expression of transcripts involved in TGF-β, immune cell recruitment and coagulation, and a reduction in a key regulator of EC barrier function, β-catenin signaling. Changes in the nuclear levels of TDP-43 in the endothelium are sufficient to drive many of the transcriptional alterations observed in the REV1 population of capillary ECs, strongly suggesting that a disease-specific loss of nuclear TDP-43 from ECs drives loss of BBB integrity observed in AD, ALS and FTD. In support of this, our group showed that even partial loss of TDP-43 from brain endothelium, caused by heterozygous ALS/FTD associated mutation, leads to endothelial dysfunction and BBB breakdown^41^.

### Impaired coordination of NF-κB and TDP-43

The discovery of elevated nuclear p65/NF-κB levels in both cognitively healthy and neurodegenerative brain ECs highlights a paradox. In epithelial cells and ECs, NF-κB seems to be critical for barrier maintenance, a role diametrically opposed to its canonical function in inflammatory responses and barrier disruption. In ECs, nuclear translocation and transcriptional activation by NF-κB forms the core of the inflammatory response to pathogens and cytokines, culminating in increased leukocyte recruitment and permeability^44^; however, it is also apparent that some basal level of NF-κB signaling is required to maintain tight barriers. Deletion of Nemo or p65 subunits, both key components in NF-κB signaling, results in increased intestinal barrier permeability, and NF-κB inhibitors disrupt tight junctions and increase lung epithelial cell permeability doses, which did not induce cell death^45,46^. A similar role seems to exist in brain ECs, where loss of a critical component of the NF-κB signaling pathway, Nemo and Tak1, lead to BBB permeability, EC death and vascular rarefication^47,48^. Consistent with our results, high levels of basal p65/NF-κB were also observed in murine brain endothelial nuclei^19^. However, these high basal levels of nuclear p65/NF-κB somehow seem to not activate canonical downstream transcriptional responses, which are low in the murine brain endothelium^49^ and in our isolated human endothelial nuclei. The molecular mechanisms underlying this paradox have been unclear.

Our research sheds light on this paradox, particularly regarding the significant impact of TDP-43 disruption on brain ECs. Comparative analysis of healthy and diseased endothelial nuclei reveals a unique pattern in the diseased groups. While the spectrum of nuclear p65/NF-κB levels is comparable in both groups, only the diseased group demonstrates a pronounced canonical NF-κB response. As this correlates with a drop in the levels of nuclear TDP-43 relative to NF-κB, we propose that TDP-43 limits NF-κB-mediated activation of canonical transcriptional responses while permitting noncanonical functions critical for BBB maintenance. Our data from targeted disruption of TDP-43, in vivo and in vitro, support this concept. We observe increased levels of canonical TNF/NF-κB targets in ECs with deletion or suppression of TDP-43. Thus, we propose that TDP-43 levels rise with nuclear levels of NF-κB, and that this relationship must be maintained to allow NF-κB to maintain the BBB without triggering transcriptional activation of well-known proinflammatory pathways. Our data show that this relationship is disrupted in AD, ALS and FTD.

### Reduced Wnt/β-catenin in neurodegeneration

The Wnt signaling pathway plays a crucial role in the formation and maintenance of the BBB; however, its role in the progressive barrier dysfunction in neurodegenerative disease has not been explored in human tissues^50^. Although changes in Wnt signaling have been observed in the brain tissue in AD, it is not clear whether this leads to altered Wnt signaling in ECs. Here, using both a protein-based readout of nuclear β-catenin and a transcriptional readout of Wnt signaling activity in the same cells, we show a reduction in both measures in a subset of brain ECs associated with AD, ALS and FTD. A possibility is that it is linked to TDP-43 loss. Cells with targeted deletion or suppression of TDP-43 in vitro or in vivo showed a similar increase in Wnt-suppressed transcripts and decrease in Wnt-induced transcripts^41,42^. Future work focused specifically on these signaling pathways in tractable animal models will be required to delineate these interactions.

### TDP-43 and the mechanical forces of blood flow

Loss of TDP-43 may be sufficient to interfere with the cellular machinery downstream of physiologic laminar flow. As the presence of flow provides homeostatic cues to the endothelium^51–53^ to limit proliferation and apoptosis, to reduce transcriptional output from p65/NF-κB^54^, and to enhance brain barrier function^55,56^, disruption of these signaling pathways is predicted to lead to endothelial and BBB dysfunction. While the underlying mechanism is not yet clear, our data show that even a single-allele disease-associated mutation of Tardbp is sufficient to affect the cellular cytoskeleton and continuous tensegrity with the extracellular matrix. These mechanical connections are the basis of flow-mediated responses in the endothelium^51^, and their disruption is predicted to impair homeostatic flow responses. While it is presently unclear how the level and function of TDP-43 modifies the cytoskeleton and these responses, this may be key to understanding BBB disruption in neurodegeneration.

In summary, we identify a proinflammatory, barrier-compromised endothelial subtype in neurodegenerative diseases AD, ALS and FTD. In these cells, nuclear TDP-43 is reduced. This is sufficient to increase canonical p65/NF-κB transcriptional activity and disrupt Wnt/β-catenin signaling. A deeper understanding of the molecular mechanisms in these ECs is likely to provide improved biomarkers for early vascular alterations and strategies to mitigate barrier and vascular dysfunction in neurodegeneration.

## Methods

### Human tissues and regulatory oversight

Cortical samples from Brodmann Area 10 were obtained from the NIH NeuroBioBank. We thank the donors and families for their contributions to the scientific research reported here. Samples were obtained from the Maryland branch of the NeuroBioBank, and under a protocol that has been reviewed and approved annually by the University of Maryland Baltimore Institutional Review Board. No compensation was provided. The cohort included a total of 92 donors, comprising young donors (*n* = 20; 10 male and 10 female; aged 15–29 years) and aged donors (*n* = 20; 14 male and 6 female; aged 67–95 years) without neurodegenerative conditions, as well as donors with neurodegenerative diseases: AD (*n* = 24; 11 male and 13 female), ALS (*n* = 20; 11 male and 9 female) and FTD (*n* = 8; 4 male and 4 female). Further details on the de-identified samples are provided in Supplementary Fig. 1 and Supplementary Table 1. Three donors with discrepancies between biobank disease classifications and patient records were excluded: one sample due to concurrent conditions that could affect endothelial function, such as familial hypercholesterolemia; another because neuropathology reports indicated normal findings, inconsistent with the medical diagnosis of ALS; and a third because patient records indicated Lewy body dementia.

### Statistics and reproducibility

No statistical method was used to predetermine sample size. Samples sizes were not calculated by power analysis, but were defined by logistical limitations of sample availability and sequencing costs. Generally, samples sizes expanded significantly beyond previous published work on cerebrovascular endothelium (Supplementary Fig. 4). To enhance reproducibility and ability to detect differences, enriched endothelial nuclei samples were combined such that single 10x reactions contained both disease and healthy donors, allowed for normalization by 10x reaction without requiring donor-specific normalization. The experiments were not randomized and the investigators were not blinded to allocation during experiments and outcome assessment, as sample allocation was predetermined and approaches were general and quantitative. Data distribution for gene expression and gene-gene correlation was assumed to be normal but this was not formally tested. Gene set enrichment approaches are nonparametric and no assumptions are made. In addition to standard approaches to differential gene expression, the incorporation of a large number of samples facilitated pseudobulk analysis to limit the effects of single biological outliers.

### Antibody–oligonucleotide conjugation

Generally, conjugation followed published protocols^57,58^, with some minor modifications. The purchased antibodies (Supplementary Table 2) were generally BSA- and azide-free. If antibodies included BSA as a stabilizer, they were cleaned with Melon columns according to the manufacturer’s protocol (Thermo, 45206). Antibodies were cleaned twice using a pre-wet Amicon Ultra-0.5 30-kDa MWCO filter, as suggested in the protocol. Subsequently, the columns were inverted and 5 µl diluted 4 mM mTz-PEG4-NHS was added, followed by a 30-min incubation for antibody functionalization. To quench the reaction, 1 µl 1 M glycine (pH 8.5) was added. Any excess mTz-PEG4-NHS was removed by filtering through the same 30-kDa MWCO filter for 5 min at 14,000*g*. The filter was then inverted into a clean collection tube and spun at 100*g* for 2 min to elute the product. The functionalized antibody was subsequently allowed to react with TCO-PEG4-oligonucleotide. For every 1 µg of antibody, 30 pmol TCO-PEG4-oligonucleotide was added. The reaction mixture was then incubated overnight in a 4 °C fridge. After the reaction was complete, one-tenth of the reaction volume of 10 mM TCO-PEG4-glycine was added to quench any residual tetrazine reaction sites on the antibody. Conjugation was verified using western blot analysis, which checked for an increase in the size of the heavy chain after conjugation and laddering for the hyperconjugated product (Supplementary Fig. 17). To eliminate unbound excess oligonucleotides, the reaction mixtures of individual antibody/oligonucleotide pairs were combined and subjected to two ammonium sulfate precipitation steps for purification. The resulting elutes containing antibodies were applied to a size exclusion chromatography column (Superdex 200 10/300 GL; GE Healthcare) with PBS served as running buffer and a flow rate of 0.5 ml min^−1^ (Supplementary Fig. 17). The elution of the factions was monitored simultaneously at wavelengths 280 nm and 260 nm using an AKTA pure chromatography system equipped with a UV monitor U9-M (Supplementary Fig. 17). The list of conjugated antibodies is shown in Supplementary Table 2.

### Enrichment of endothelial nuclei from frozen postmortem brain tissue and inCITE-seq antibody labeling

Nuclei isolation generally followed our previously published protocol with minor modifications^18^. All steps were performed either on ice or at a temperature of 4 °C, unless specified otherwise. Frozen cortical brain tissues, weighing 100–200 mg, were thawed at room temperature for 3–4 min in Nuclei EZ lysis buffer (Sigma, Nuc101) mixed with RNase inhibitor (0.5 U µl^−1^, Clontech, 2313) in RINO tubes containing eight 3.2-mm stainless steel beads. The tissues were then mechanically homogenized using a Next Advance Bullet Blender BB724M, set to level 4 for 4 min at 4 °C. Following homogenization, the mixture was diluted in 5 ml, centrifuged at 500*g* for 5 min (retaining the supernatant for analysis of total brain protein and messenger RNA) and subsequently washed with 5 ml Nuclei EZ lysis buffer. A 2-min incubation on ice preceded a second centrifugation at the same speed for another 5 min. The supernatant was then discarded, and the homogenate was resuspended in 5 ml Nuclei EZ lysis buffer, followed by a 5-min incubation on ice. The homogenate was then strained through a 70-µm pluristrainer, centrifuged at 4 °C and the supernatant removed. The resulting nuclei pellet was suspended in 200 µl of a PBS-based staining solution (1% BSA, PBS and 0.5 U µl^−1^ RNase inhibitor) in 1.5-ml low-bind tubes. After washing the nuclei with staining buffer (PBS + 0.2% BSA + RNase inhibitor), the supernatant was removed and 50 µl blocking buffer, containing ssDNA (1 mg ml^−1^ final concentration) and FC block (1:100 dilution, BioLegend, 156604), was added. This was followed by a 10-min incubation. Before using the antibody mix, we incubated it with EcoSSB (Promega M3011) in 50 μl 1× NEBuffer 4 for 30 min at 37 °C as suggested previously^20^. The nuclei were then stained with a buffer containing anti-Erg 647 (1:200 dilution, Clone EPR3864, Abcam), anti-NeuN Cy3 (1:200 dilution, Sigma), 4,6-diamidino-2-phenylindole for nuclei labeling (1:2,000 dilution of a 5 mg ml^−1^ stock) and inCITE-seq antibody mix (1:400 dilution of concentrated oligonucleotide-conjugated antibody mix). Afterwards, 1 µl TotalSeqB Hashtag (anti-Nuclear Pore Complex Hashtag at 0.5 g, BioLegend 682239-682243) was added to each sample for subsequent identification in downstream analysis. After staining, the nuclei were washed twice with staining buffer and fixed with 2% PFA in staining buffer for 1 min at 4 C. Then, 500 µl staining buffer containing 0.1% glycine was added to quench the fixation. The nuclei were centrifuged for 5 min at 500*g* twice and resuspended in 500 µl staining solution. This solution was then filtered through a 35-µm filter before sorting the nuclei using an Aria 2 with a 40-µm nozzle, collecting them into a BSA-coated tube maintained at 4 °C.

### Droplet-based 10x genomics single-nucleus RNA sequencing

For human tissues, nuclei from 4 to 5 human donors were pooled to a final volume of 42 µl. We aimed for each 10x reaction to include two control groups (one from an older healthy individual and another from a younger individual) and two groups representing different disease states (AD, ALS or FTD), each uniquely tagged with a nuclear pore hashtag for subsequent identification using DemuxEM. We aimed for total nuclei count in each mixture to be ~8,000 nuclei. These mixtures were then loaded onto a Chromium single-cell V3 3′ chip (10x Genomics) and processed according to the manufacturer’s protocol. In brief, after GEM generation, they were subjected to reverse cross-linking and reverse transcription by heating at 53 °C for 45 min and then at 85 °C for 5 min. The samples were subsequently stored at 4 °C until GEM recovery.

For cDNA amplification, the standard 10x Genomics protocol was used, adding Feature cDNA primer 2 into the mix. Following amplification, an SPRI-based size selection was carried out to separate antibody–oligonucleotide-derived cDNA from mRNA-derived cDNA. The antibody–oligonucleotide cDNA, contained in the supernatant, was set aside for antibody capture library construction. Meanwhile, the mRNA-derived cDNA, attached to SPRI beads, was processed for gene expression library construction. Gene expression libraries were prepared from the mRNA-derived cDNA using enzymatic fragmentation, adaptor ligation and sample indexing, following the 10x Genomics protocol. These libraries were stored at −20 °C until quantification and sequencing. For antibody capture library, the antibody–oligonucleotide-derived cDNA underwent mixing with 1.4× SPRIselect, magnetic separation and ethanol washes, followed by elution in sterile water. PCR amplification of the cDNA with distinct TotalSeq-B index primer mixes generated the protein capture libraries. Subsequently, these libraries underwent purification, elution, and were stored at 4 °C. Quantification and sequencing were performed on a NovaSeq, utilizing v.1.5 chemistry.

### Data processing

The key processing steps are listed in https://github.com/pamurphyUCONN/2024_Omar.

### inCITE-seq and snRNA-seq data preprocessing

Sequencing data were processed with Cell Ranger v.7.0.1 on Xanadu high performance computing cluster (UConn Health Center). Reads from FASTQ files were aligned to mouse mm10 or human GRCh38 reference genome as described by 10x genomics. Paths to feature library (antibody capture and gene expression) were specified during processing, so we ended with an h5 object that included both gene expression and antibody capture information. Hashtagged nuclei were demultiplexed using DemuxEM using Pegasus^59^ implementation in Python^60^ with parameters min_signal = 15.0, α = 0.0, α_noise = 150.0 and deMULTIplex2 (ref. ^61^). Nuclei with ambiguous hashtag assignment were discarded. All downstream analyses were performed using Scanpy (v.1.8.1)^62^. The dataset comprises 47 human samples featuring both RNA and protein capture, while the remainder includes only RNA analysis (Supplementary Table 1). Protein data from these samples were excluded from RNA-only samples due to insufficient antibody counts, rendering them unsuitable for reliable protein analysis. This issue was addressed in subsequent experiments by increasing the antibody concentration at ~0.5 µg of each antibody per sample.

### snRNA sequencing analysis

Nuclei were required to express at least 50 genes and each gene had to be present in a minimum of five cells. Nuclei were excluded if they exhibited more than 5% mitochondrial gene content, had nuclear pore hashtag counts exceeding 4,000 or displayed anti-TDP-43 antibody levels above 3,000 antibody counts. The normalization process for gene counts in each nucleus involved individual normalization followed by logarithmic scaling using the formula ln(x + 1).

For cell type clustering (Fig. 1) 6,830 variable genes were identified using the ‘highly_variable_genes’ function in Scanpy, with the parameters set to min_mean = 0.0015, max_mean = 0.18, and min_disp = 0.30. The log counts were scaled and unique molecular identifier counts were adjusted using the ‘regress_out’ function in Scanpy. Dimensionality reduction was then carried out on these variable genes using PCA in Scanpy, employing the arpack solver. To account for batch effects, Harmony was applied^9^, focusing on corrections for 10x well batches and individual human samples effects. Then, a *k*-nearest-neighbor graph was constructed using the top 40 principal components and *k* = 10 neighbors, followed by clustering using the Leiden algorithm at a resolution of 0.8. The data were then visualized using UMAP. Further, 9,169 suspected doublets were removed using scrublet. After removal of those nuclei, PCA and Harmony-corrected PCA were recalculated to account for reduced number of nuclei. Cell types were then annotated using the Pegasus annotate function. Nuclei with indeterminate assignments were removed, resulting in 132,859 cells.

For disease-specific clustering (Figs. 3–6), as clustering to define cell types used Harmony to limit variation due to disease state, we aimed to return to major cell type classifications derived by original clustering to specifically examine similarities in transcriptional states between disease states and donors. We focused on two major groups, capillary ECs and microglia. For each, nuclei associated with this cluster were isolated in silico and then re-clustered, this time regressing out only effects of the 10x well batch but not disease state or donor.

Pseudobulk profiles were generated on a per-patient basis using the Scanpy implementation of the decoupleR suite with the ‘get_pseudobulk’ function. An AnnData object was generated using patient sample IDs as observations and the sum of raw gene counts for each cell type. The data were then filtered to obtain at least ten cells per cell type per donor and counts per donor per cell type of at least 800. After generating all the AnnData objects, the DESeq2 implementation in Python was run on all AnnData objects. We compared all states used in this study: AD, ALS, FTD and young to aged controls as a ref. ^63^. In the run_deseq function, covariates of batch data were corrected for. Downstream GSEA on pseudobulk profiles was carried out as described in detail below.

### Protein capture normalization

We normalized the protein amount as previously reported, except that we normalized to H3 histone instead of nuclear pore protein hash^19^. This change was made because the nuclear pore protein hash showed greater variability than H3 histone across different disease states and nuclei (Supplementary Fig. 8). This normalization was achieved using the formula: *n*(antibody capture counts) = (antibody capture counts)/histone H3 counts. Following this, we transformed these normalized values into centered natural log ratios, termed ‘nCLR’, using the formula nCLR = *n*(protein_capture)/ (∏in(protein_capture)i^(1/n)^. Here, the denominator represents the geometric mean, calculated by taking the nth root of the product (∏) across each nucleus (i). The normalized protein data were visualized using density plots, which were representative of the quintiles of protein expression. We predefined these quintiles and labeled each individual cell observation with the quintile range it fell into. For further analysis, we employed the embedding_density function from the Scanpy tools suite. This function was used to calculate an embedding density protein, which was then visualized using the embedding_density plotting function. Relating gene expression to TDP43 protein levels was performed using a two-step mixed-effects linear model on capillary EC clusters, as described previously^19^. In this analysis, we regressed out the effects of unique molecular identifiers, hashtag counts and batch using the formula gene ~ log_ncounts + log_hashtag_counts + C(date). The residuals for each gene from the first step to model the effect of TDP43 and other relevant proteins, like P65 and β-catenin. The model was run on genes expression in at least 25 nuclei using layers of the data. The significance of the associations between gene expression and protein levels was determined using an FDR of 0.05, with correction for multiple testing applied via the Benjamini–Hochberg method. The get_significance_df function^64^ was used to compute the significance based on the adjusted *P* values. To assess the overlap between genes associated with TDP43 and TDP43 targets identified by CLIP-seq from control brain tissue^65^, we compared genes with positive or negative coefficients and performed a Fisher’s exact test to determine the significance of the overlap.

### Pathways and gene set enrichment analysis

We identified marker genes and DEGs between clusters using the *t*-test method, as implemented in the rank_genes_groups() function in Scanpy. For microglia pathway analysis, we utilized the PROGENy database implemented in Python^66^. Specifically, we exported the top 1,000 terms from the PROGENy database. We then compared the inferred Leiden cluster pathways with inferred cellular activity on a per-cell basis using the run_mlm function. The results were saved in the obsm of the AnnData object and visualized through a heatmap and on a UMAP to highlight differences in specific inferred pathways. Genes downstream of the pathways in PROGENy were visualized using a scatter-plot, plotting the weight from the PROGENy table on the *x* axis against log fold changes from the differential gene expression analysis. Enrichment and GSEA were performed using the exported differential gene expression table. For NF-κB-related pathways, we utilized the curated Hallmark gene sets from MSigDB. For β-catenin analysis, we employed custom-curated gene sets derived from studies on β-catenin function modulation in pluripotent stem cell-derived ECs^67^. In our cell proportion analysis, we assessed the relative contribution of disease states in clusters to identify common associations in gene expression (GEX) in dementia. This analysis involved visualizing the relationship between age and the proportion of cells in each cluster using scatter-plots with regression lines. We quantified this relationship using Spearman’s rank correlation.

### GSEA of similarities in gene expression patterns

To examine overlap in gene sets, we used GSEApy. In brief, we generated a pre-ranked list (based on log_2_ fold change) of DEGs (*P* < 0.05) between healthy and disease capillary clusters from our inCITE-seq data. We generated similar pre-ranked lists from our data on experimental deletion or silencing of TDP-43 in human or mouse brain ECs, or from cells of mice with ALS/FTD associated mutations in Tardbp or Grn. A full list of these datasets is found in Supplementary Table 3 (and the SRA datasets found at PRJNA1054818). Differential gene expression was performed by DESeq2, using RSEM-generated gene counts after STAR alignment as described^41^. These ranked lists were filtered for BaseMean expression > 5 to exclude genes that were not expressed at a sufficient level to be called as differential and ranked based on log_2_ fold change. We then examined the relative enrichment of MSigDB Hallmark (2020) and KEGG (2021) pathway gene sets within the ranked list, using GSEApy parameters ‘log_2_ ratio of classes’ and ‘weighted score type 1’ with 1,000 permutations. In addition, we created custom gene sets from our own data or from publicly available data. The specific datasets used and their parameters are listed in Supplementary Table 3. From these datasets, we extracted the most significantly upregulated or downregulated genes, resulting in the generation of two gene sets from each perturbation (Supplementary Table 4). These custom gene sets were mapped to the human homologue using BioMart if derived from mouse data, and then used in a GSEA search as previously described. A NES and adjusted *P* value resulting from these GSEA searches are shown on the associated heatmap. To determine similar pathways, the overlap in pathways is shown in NetworkX, where the overlap between any two datasets is given as the total number of genes in common between the datasets divided by the total number of genes in both datasets (% overlap). Key enriched pathways were plotted in GSEApy, to show the placement of pathway genes among the up- or downregulated transcripts in the datasets.

### Western analysis of protein supernatant from nuclei isolation

After centrifugation to spin nuclei down from EZ lysis buffer, samples were stored at −80 °C. Protein quantification was performed by BCA assay (Thermo, 23225). A total of 20 μg protein was loaded in 1× Laemmli loading buffer with dithiothreitol reduction, heated for 2 min at 85 °C and loaded on a Tris glycine SDS–PAGE gel (4–20%, Invitrogen) along with a PrecisionPlus Kaleidoscope protein ladder (Bio-Rad, 1610375). Protein was transferred to Immobilon-P PVDF membrane on a TurboBlot station (Bio-Rad). Blots were blocked in 5% BSA block in TBS-T and stained with pTau (Invitrogen, 44752G, 1:1,000 dilution) in 0.5% BSA in TBS-T. After 2 h with rotation in a sealed bag, blots were washed in TBS-T and stained with secondary (anti-primary HRP-conjugated at 1:5,000 dilution) for 1 h. They were washed again with TBS-T and then incubated with luminescent HRP substrate (Fisher, PI32109) before imaging on a Bio-Rad ChemiDoc MP system with multiple exposures. Pre-threshold exposures were used for quantitation. The signal was measured in ImageJ using the line tool; numbers were exported to R for data analysis. The signal was normalized to the background signal on the blot outside the lane.

### Reporting summary

Further information on research design is available in the Nature Portfolio Reporting Summary linked to this article.

## Online content

Any methods, additional references, Nature Portfolio reporting summaries, source data, extended data, supplementary information, acknowledgements, peer review information; details of author contributions and competing interests; and statements of data and code availability are available at 10.1038/s41593-025-01914-5.

## Supplementary information

Supplementary Information Supplementary Figs. 1–17 and captions for Supplementary Tables 1–8.

Reporting Summary

Supplementary Table 1 Donor tissue attributes.

Supplementary Table 2 inCITE-seq antibodies.

Supplementary Table 3 Pseudobulk analysis of AD donors compared to unaffected old donors by cell type.

Supplementary Table 4 Pseudobulk analysis of ALS donors compared to unaffected old donors by cell type.

Supplementary Table 5 Pseudobulk analysis of FTD donors compared to unaffected old donors by cell type.

Supplementary Table 6 Pseudobulk analysis of young donors compared to unaffected old donors by cell type.

Supplementary Table 7 Summary of RNA sequencing data used for comparisons with TDP-43 deletion.

Supplementary Table 8 Source and composition of endothelial gene sets used for GSEA.

## Data Availability

Data processing pipelines may be found on GitHub at https://github.com/pamurphyUCONN/2024_Omar and also archived on Zenodo at 10.5281/zenodo.14679104 (ref. ^68^). Source.h5 data files produced by Cell Ranger, after BCL2fastq and Cell Ranger counts, are at https://murphylabvasculardata.cam.uchc.edu/download and on Zenodo at 10.5281/zenodo.14712707 (ref. ^69^). Filtered and annotated h5ad files produced are at https://murphylabvasculardata.cam.uchc.edu/download and on Zenodo at 10.5281/zenodo.14712288 (ref. ^70^) (all cells) and 10.5281/zenodo.14712707 (ref. ^69^) (processed capillary and microglia data). The interactive data browser can be found at https://murphylabvasculardata.cam.uchc.edu. Raw fastq data and processed data files can be found in National Institute of Mental Health Data Archive under the Collection ID 5120 ‘Contributions of Endothelial RNA-binding Protein Dysregulation to Blood Brain Barrier Defects and Neurodegenerative Disease.’ The data are available under controlled use conditions set by human privacy regulations of the NIH NeuroBioBank. To access the data, a data-use agreement is needed. This can be achieved by accessing the NDA (https://nda.nih.gov/) and requesting data access. Once access is provided, instructions will be given for data download. The process is expected to take less than a week. All other data are available on request to the corresponding author.
