# Supplementary material for: Endothelial TDP-43 depletion disrupts core blood–brain barrier pathways in neurodegeneration
Source: Nat Neurosci. 2025 Mar 14;28(5):973–84. doi: 10.1038/s41593-025-01914-5 (PMC12081287; doi:10.1038/s41593-025-01914-5)
Supplement: Supplementary file 1 — Supplementary Figs. 1–17 and captions for Supplementary Tables 1–8. [file 41593_2025_1914_MOESM1_ESM.pdf]

nature neuroscience

Supplementary information https://doi.org/10.1038/s41593-025-01914-5

Endothelial TDP-43 depletion disrupts core blood–brain barrier pathways in neurodegeneration

In the format provided by the authors and unedited

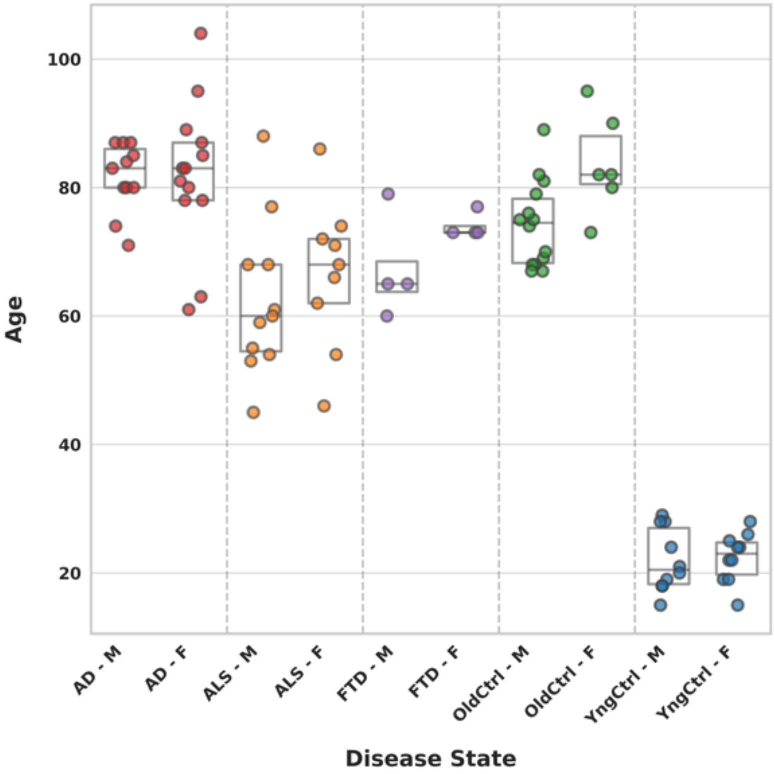

SI Figure 1. Age and sex distribution of cortical samples used.
Box plots show the age distribution of donors across disease groups (AD, ALS, FTD, OldCtrl, and YoungCtrl) and sex (M = Male, F = Female) on the x-axis, with age on the y-axis. The boxes represent the interquartile range (25th–75th percentiles) with the median (50th percentile) as the center.

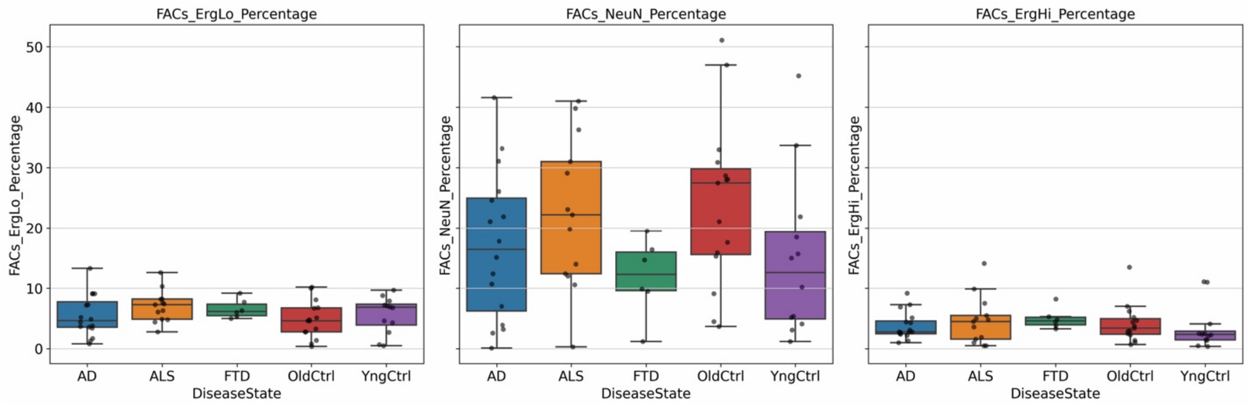

SI Figure 2. FACS populations isolated by diseases state. (A) ERGLow (microglial and macrophage) population (B) NeuN positive population and (C) ErgHi (endothelial) population. The boxes represent the interquartile range (25th–75th percentiles) with the median (50th percentile) as the center, and whiskers extending to the minimum and maximum values within 1.5 × IQR.

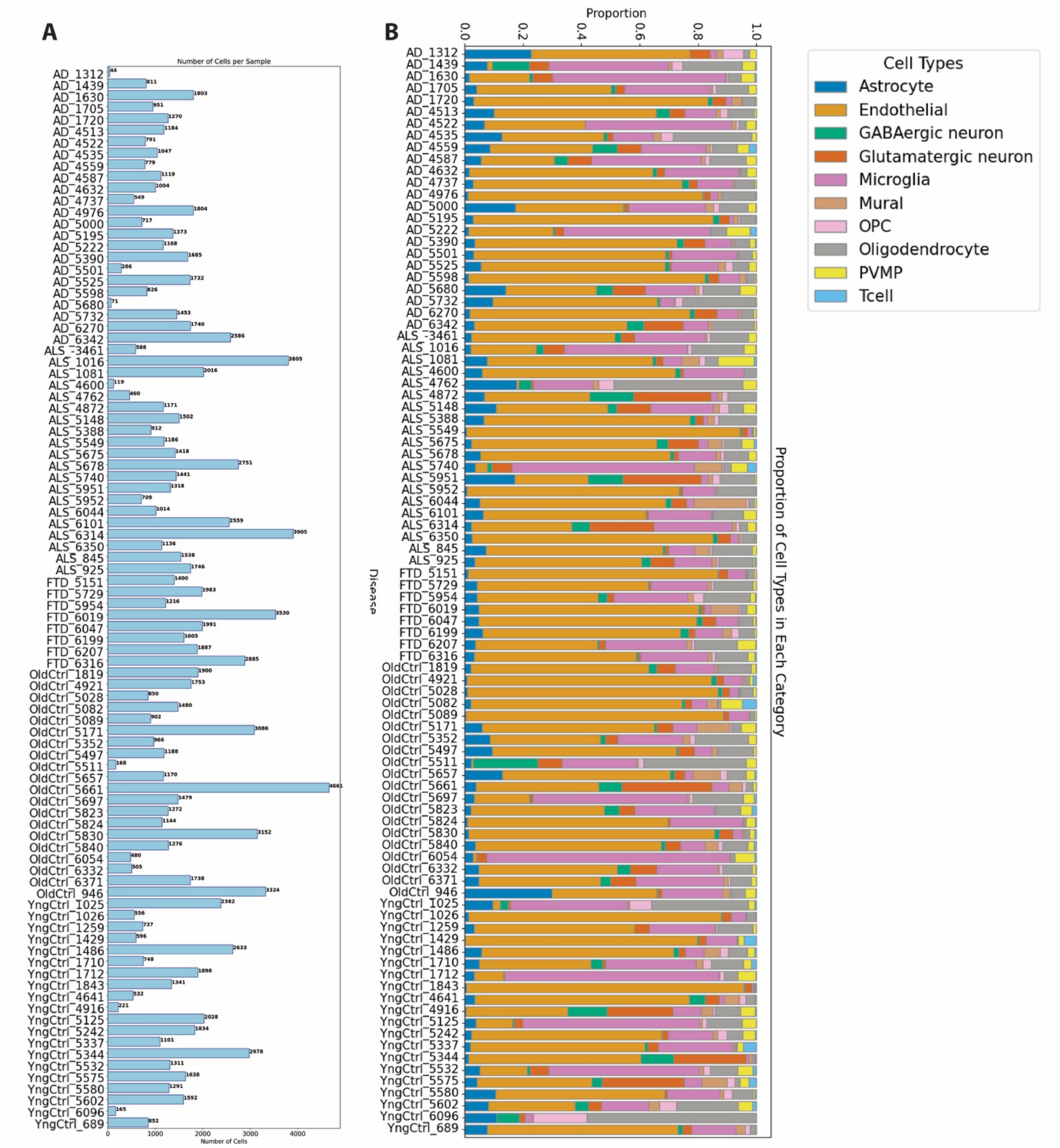

SI Figure 3. Number of nuclei and distribution of cell types in data. Sample ids are shown on the y-axis, and plots show (A) total nuclei (with count), and (B) distribution of cell types (key in top right).

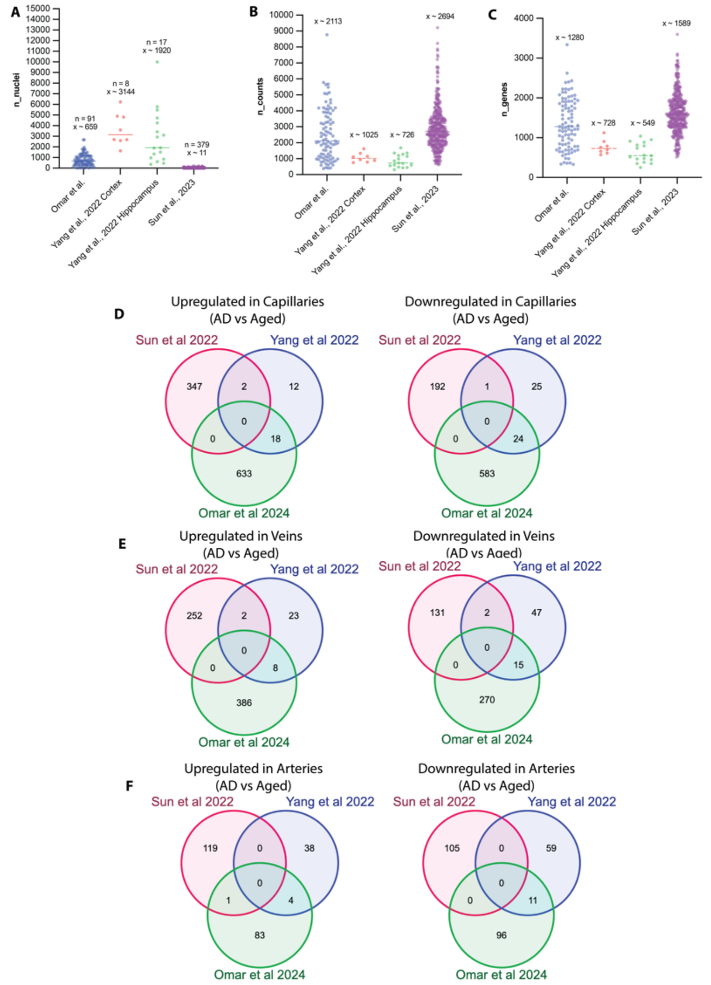

SI Figure 4. Comparison of unique molecular identifier (UMI) transcript counts and gene expression per cell and DEG overlap with prior data sets. Comparison of number of endothelial nuclei per donor (A), and number of UMI counts (B) and genes (C) per donor in data described here, relative to recently published data sets on brain vasculature and endothelium. Swarm plots are shown with individual data points representing donors and a line indicating the median for each group. (D–F) Overlap in data sets, showing differentially expressed genes (DEG, pseudobulk) between AD and unaffected older donors in the current dataset (Padj <0.05), and overlap with data sets generated by Yang et al. 2022 and Sun et al. 2022. Differentially expressed transcripts are shown as (D) increased or decreased in capillary ECs, (E) vein ECs, or (F) artery ECs.

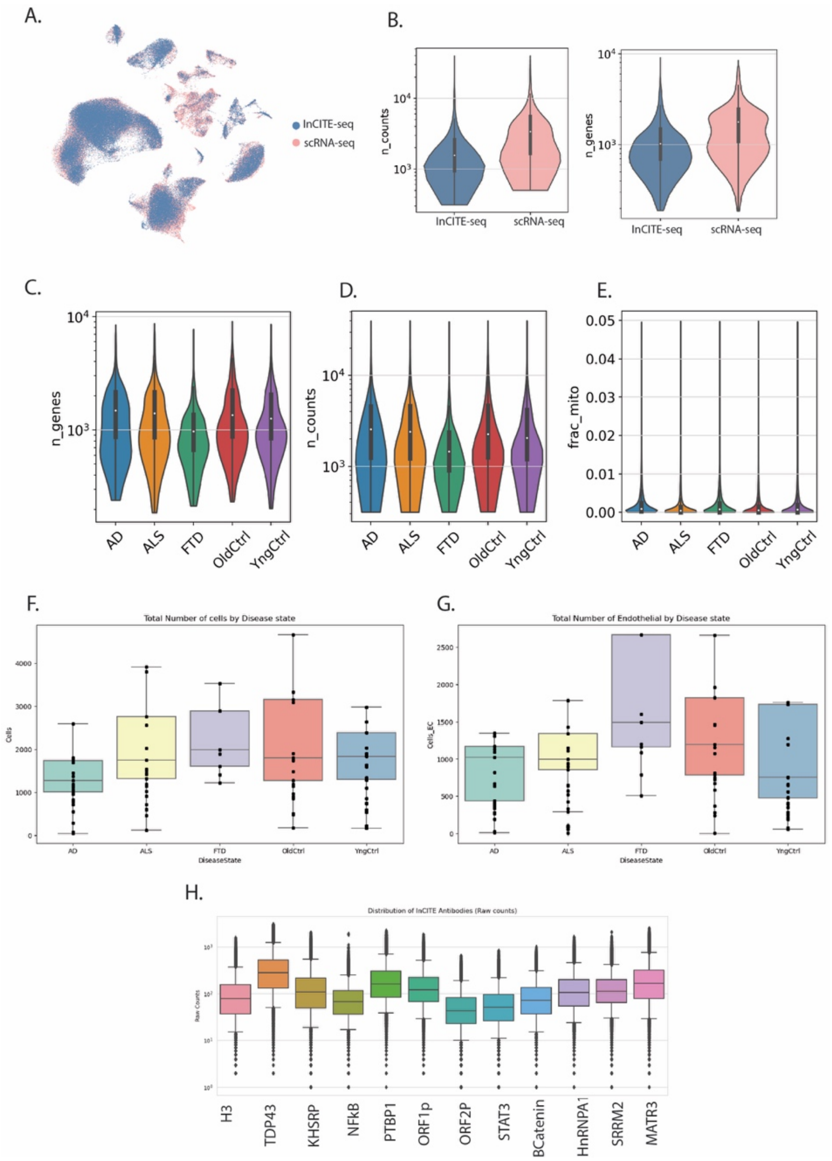

SI Figure 5. Comparative unique molecular identifier (UMI) transcript and gene counts in single nuclei data (with and without inCITE-Seq). (A) UMAP showing overlap between single nuclei data obtained from standard scRNA-Seq and inCITE-Seq preparations. (B) Violin plot of UMI counts per cell and gene counts (UMI) per cell are shown overlapped, and (C–D) by subcategory, (E) along with mitochondrial RNA counts. Inner box plots represent IQR (25th–75th percentiles) and the median (50th percentile) as the center dot. (F, G) Box plots show the (F) total number of cells and (G) ECs by disease state in each method. (H) Raw counts of CITE-Seq antibodies used in the study. Box plots represent IQR (25th–75th percentiles) with whiskers extending to 1.5 × IQR, and the median as the center line. Individual points represent outliers.

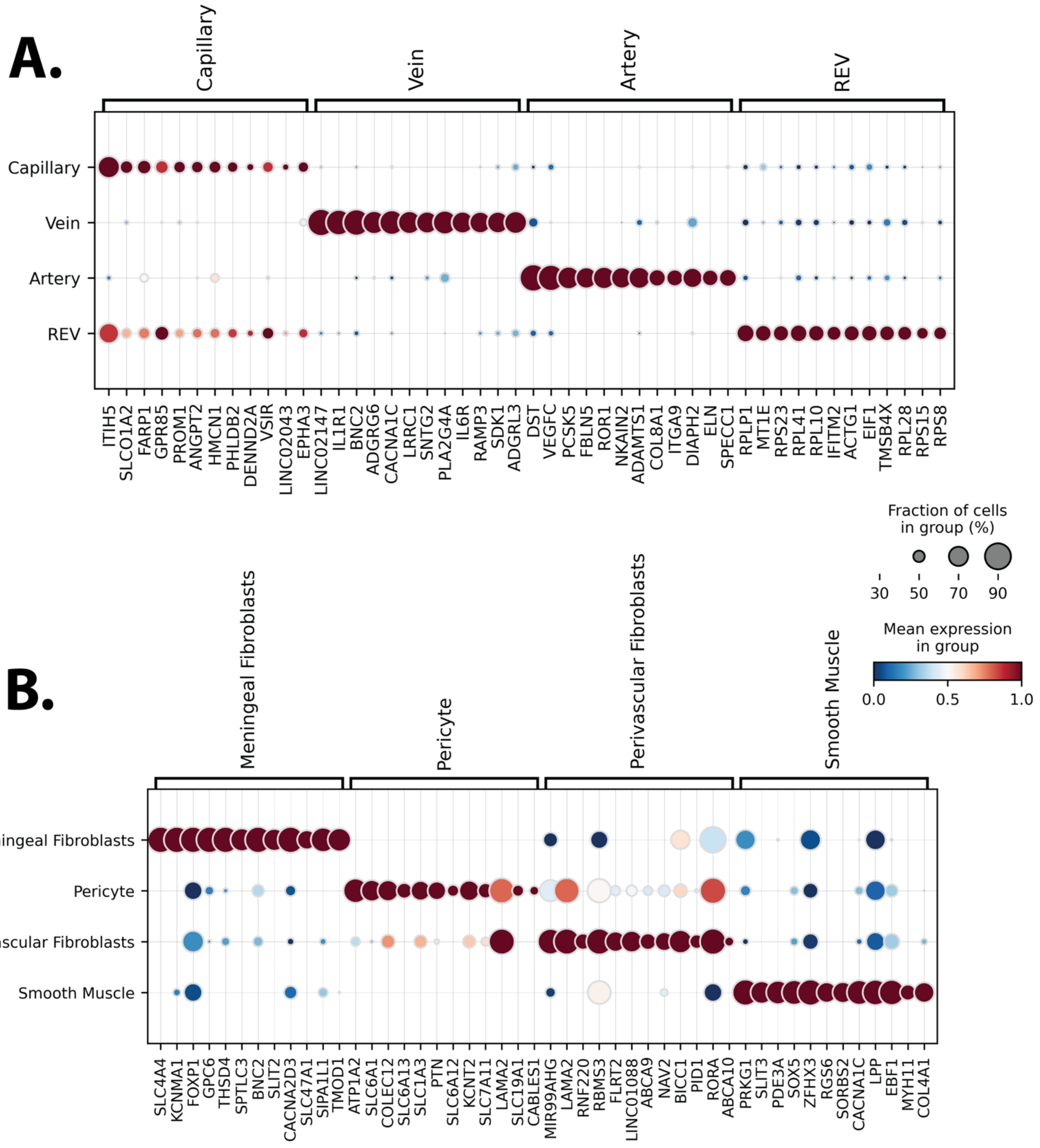

SI Figure 6. Subclustering of endothelial and mural cell types. (A) Dot plot showing top gene markers for each of the EC zonation markers for capillaries, veins, artery and reactive post capillary venule (REV) population. (B) Dot plot showing top gene markers for each of the mural cell population, including meningeal fibroblasts, pericytes, perivascular fibroblasts, and smooth muscle cells.

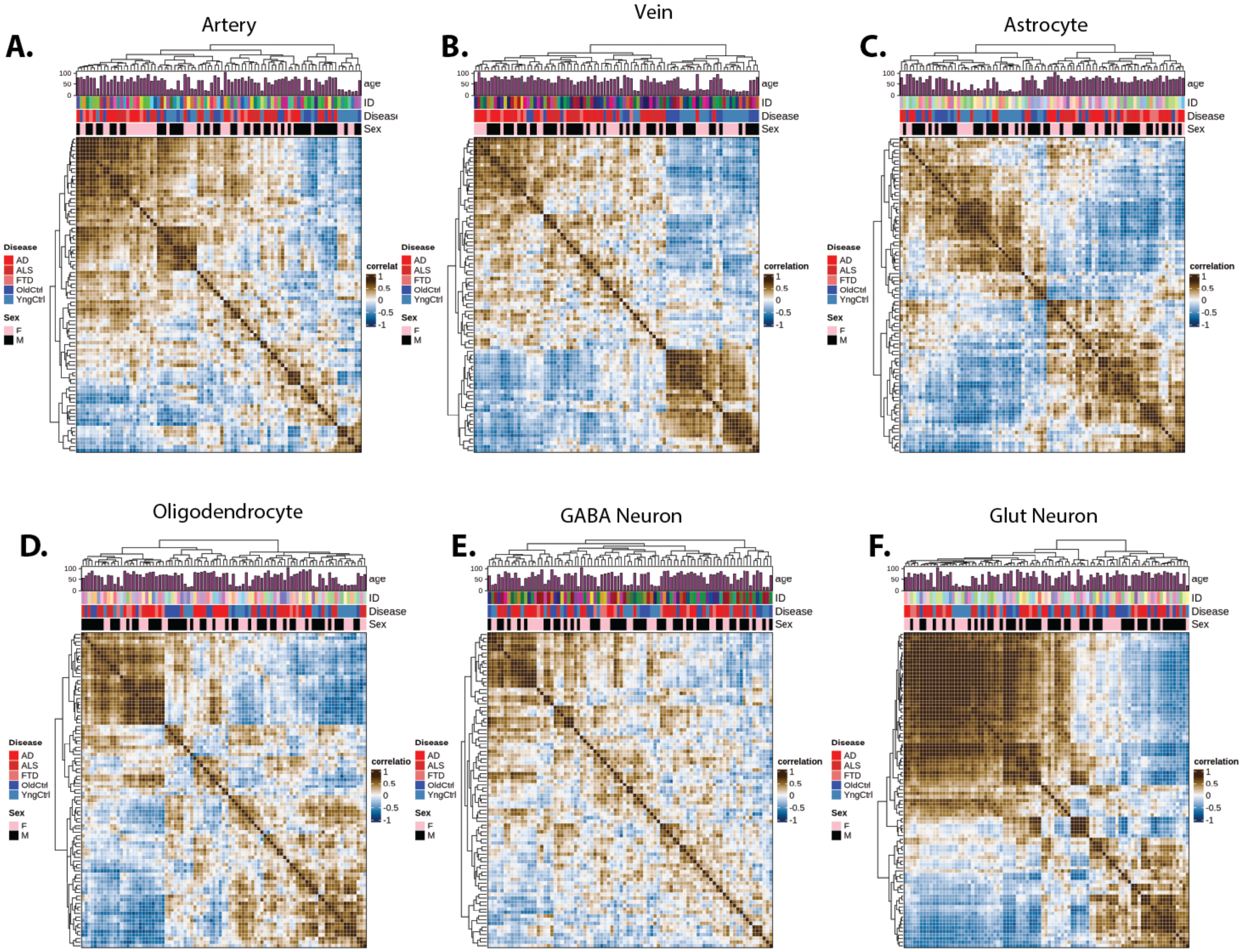

SI Figure 7. Correlation matrix by cell type and enriched transcription factor signatures.
(A–F) Correlation matrix showing Pearson correlations of PCA-adjusted cell profiles from diverse donors, processed via Harmony for integration based on batches. The PCA, based on 50 components, is followed by hierarchical clustering and dendrogram visualization, categorizing data by sex and disease state.

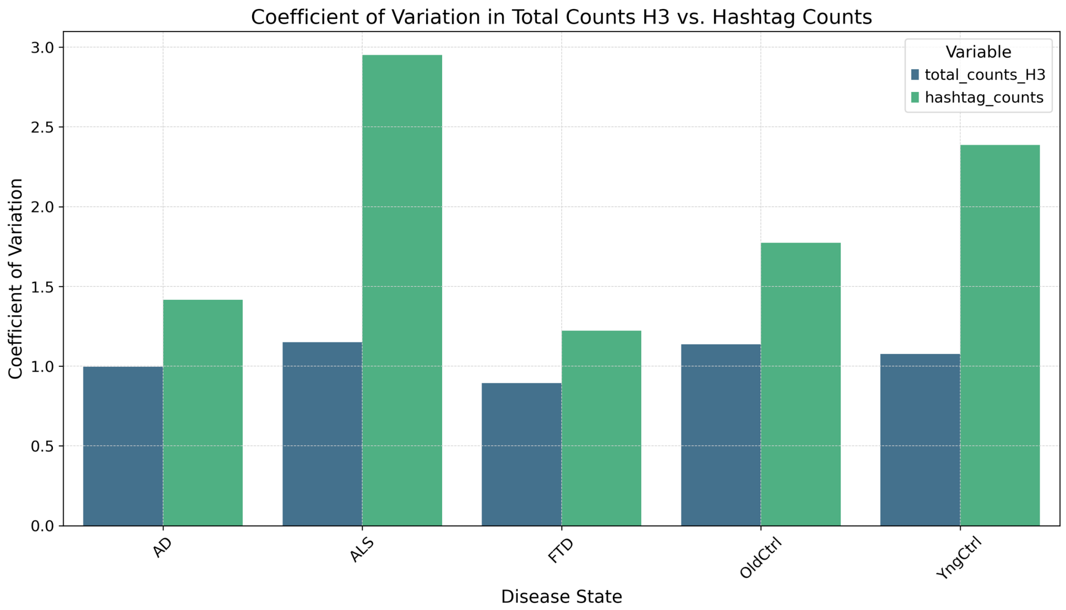

SI Figure 8. Coefficient of variation in the levels of histone and nuclear pore hashtags between nuclei by disease state. Violin plot shows coefficient of variation in levels of histone and nuclear pore hashtags across nuclei within each disease group.

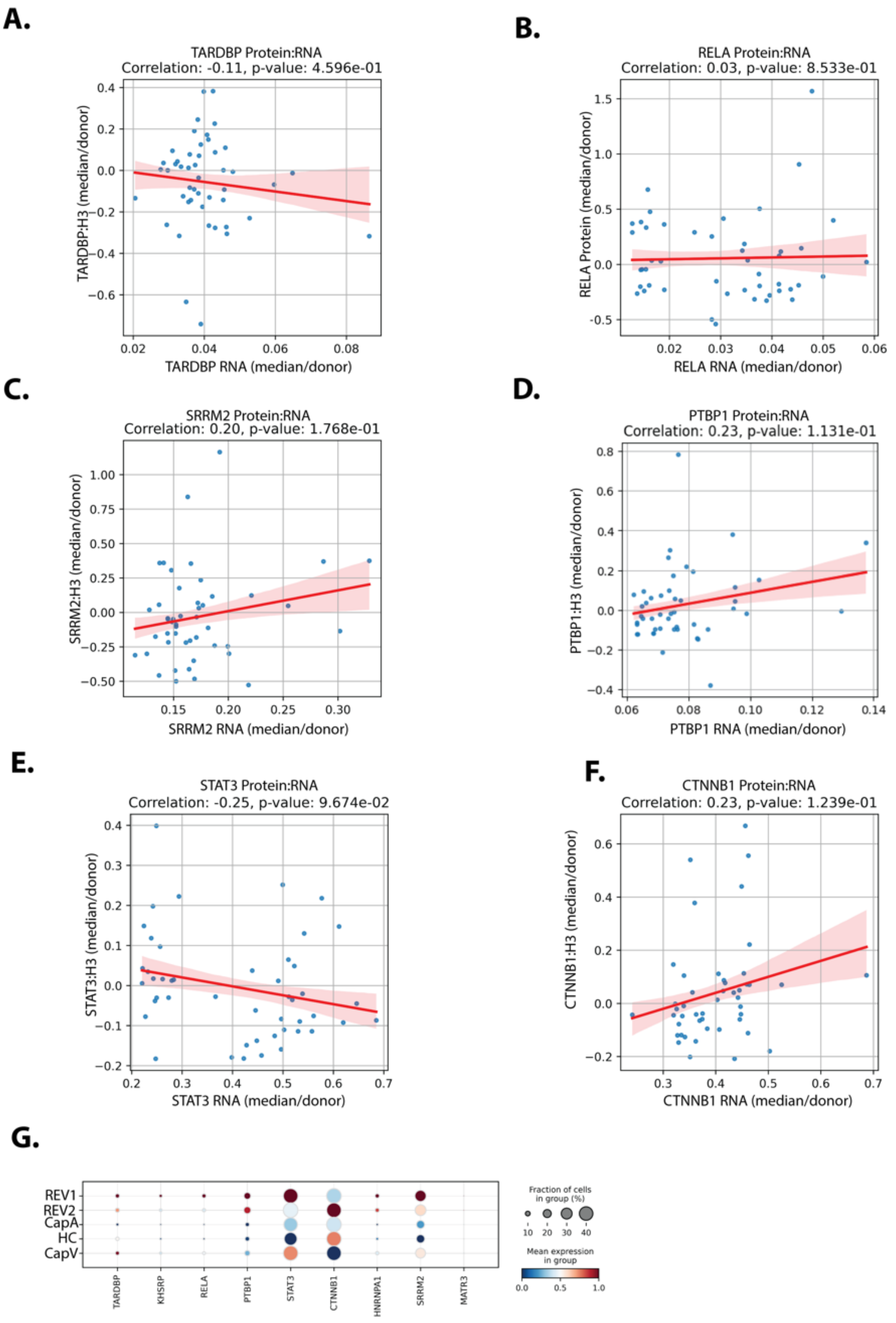

SI Figure 9. Correlation between protein and gene expression data. (A-F) Scatter plot of denoised gene expression (X-axis: median normalized gene expression per donor) with inCITE-Seq antibody normalized counts to H3 in all nuclear protein measurements used in the study (Y-axis: median normalized protein expression per donor). Correlation and P value are derived from computing two sided pearson correlation between gene expression and normalized antibody capture data per donor with 95% confidence intervals. (G) Dot plot of gene expression data for proteins used in the study within the capillary cluster.

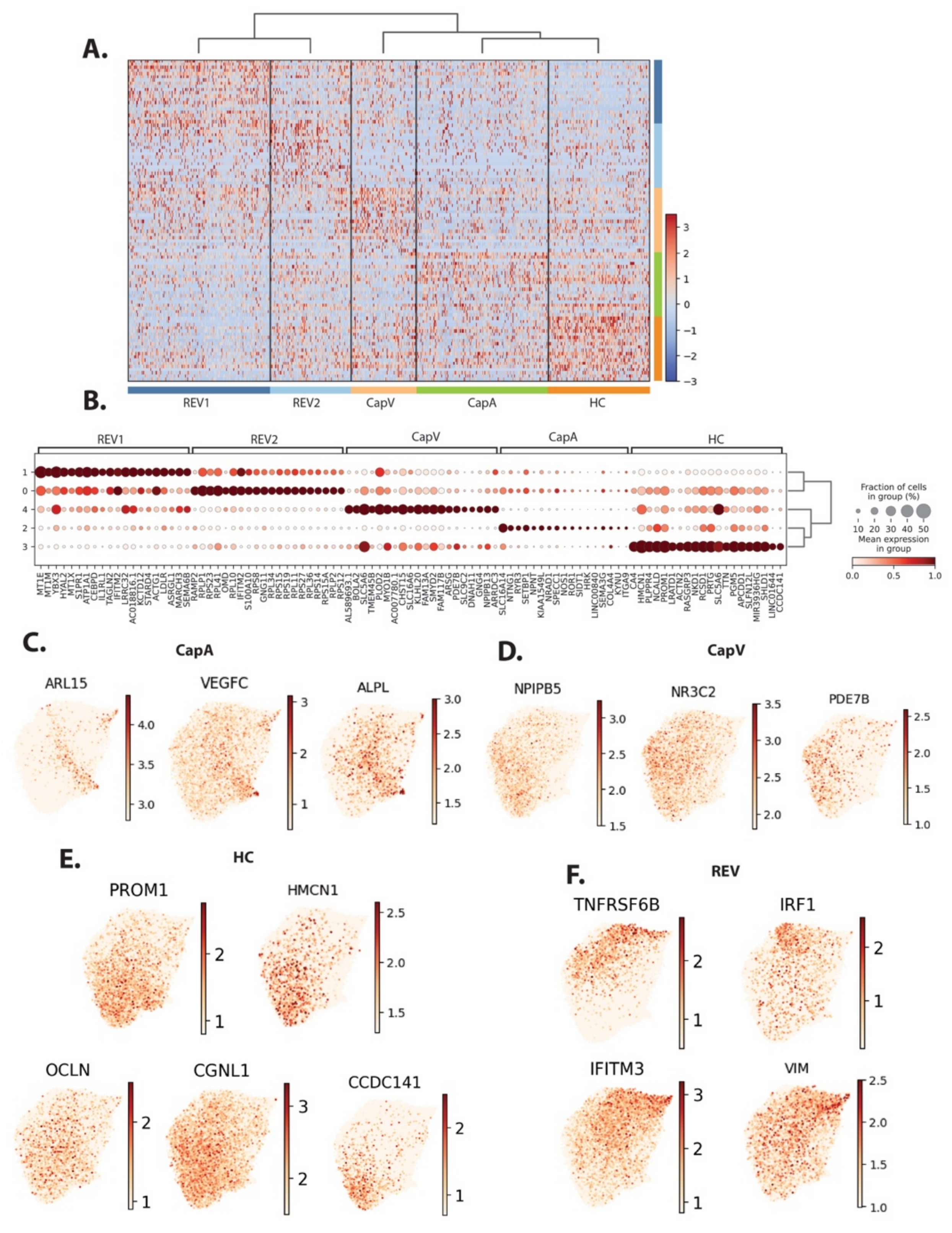

SI Figure 10. Capillary subclustering. (A) Heatmap of top differentially expressed genes in the subclusters of the endothelial capillary population. (B) Dot plot of top genes in these clusters. (C) UMAP feature plots of key markers in arteriole-like capillaries (CapA) including ARL15, VEGFC, and ALPL. (D) UMAP feature plots for venule-like capillaries (CapV) such as NPIPB5, NR3C2, and PDE7B. (E) UMAP feature plots for healthy capillaries showing genes related to cell-cell junctions including OCLN, CGNL1, and CCDC141. (F) UMAP feature plots for reactive post-capillary venules (REV) displaying inflammatory signaling genes such as IRF1, IFITM2, TNFRSF6B, and VIM.

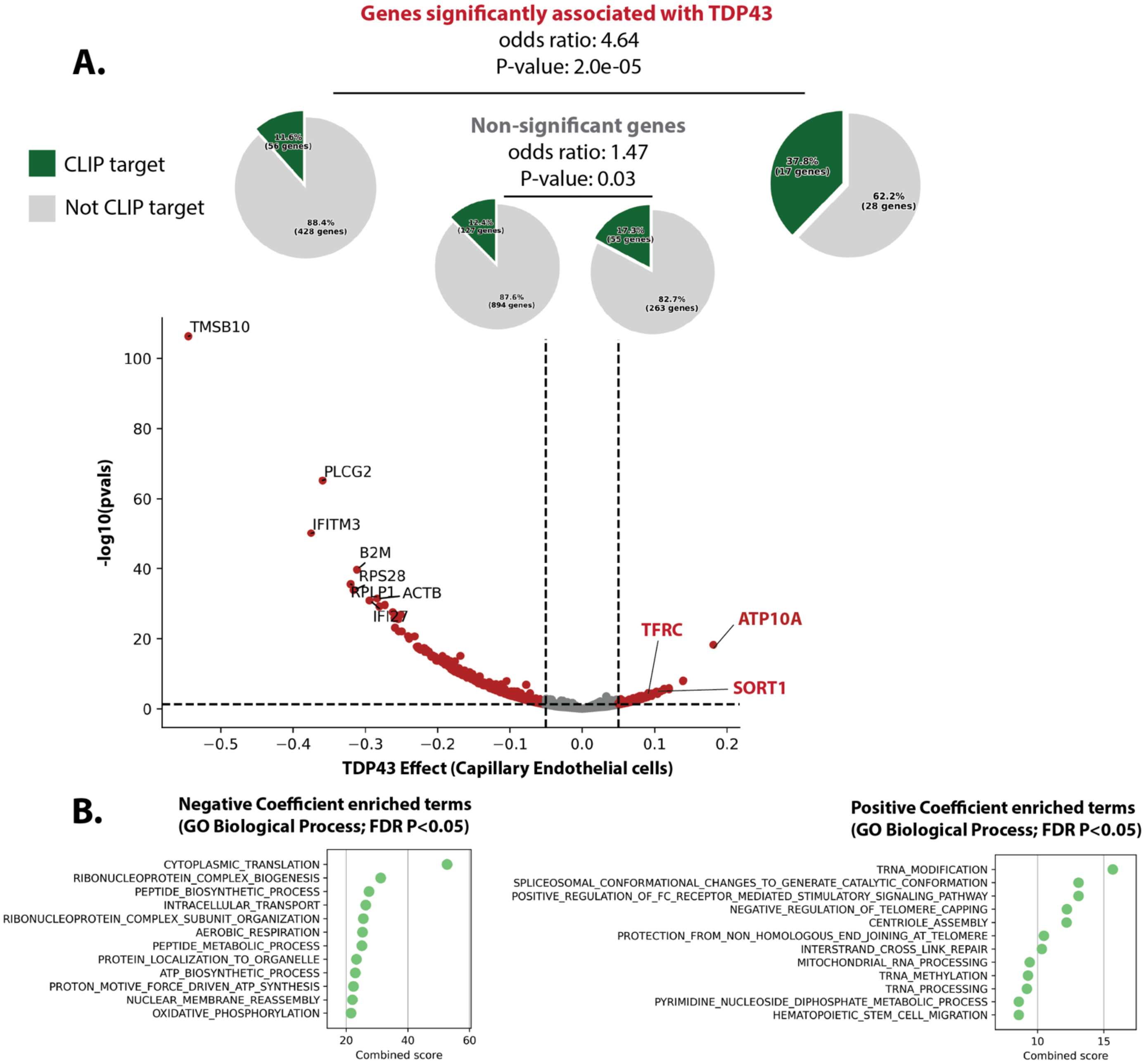

SI Figure 11. Enrichment of TDP-43 CLIP-seq targets with inCITE TDP-43 levels. (A) Volcano plot showing linear correlation of genes with TDP-43 in capillary EC cluster. Positive coefficients indicates a gene is positively correlated with TDP43 and vice versa. In pie charts, green indicates the portion of each gene set which are defined CLIP-Seq TDP-43 targets (PMID: 21358640, CLIP in unaffect brain tissue). For genes significantly correlated with TDP-43 protein levels (determined by linear modeling), the odds ratio was 4.64 with $p < 0.001$. For genes positively correlated but not significant, the odds ratio was 1.47 with $p < 0.05$. (B) Gene ontology analysis of biological processes in genes with either positive or negative coefficients (FDR P<0.05 for shown enriched pathways), combined score is derived from ENRICHR, which multiplies the z-score and p-value.

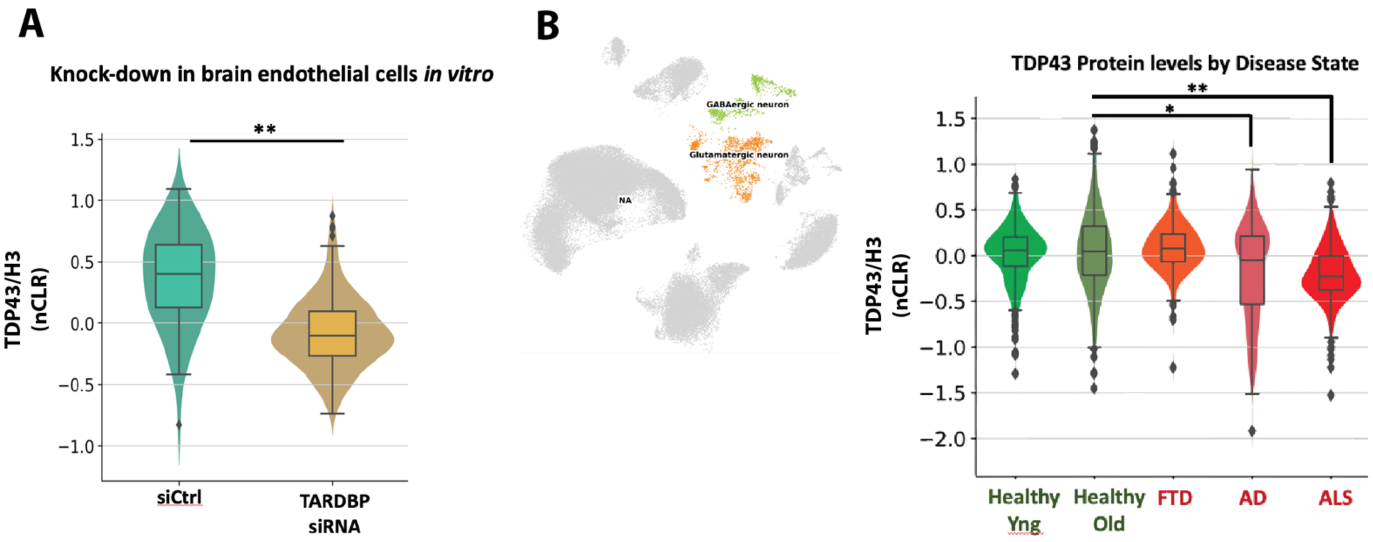

SI Figure 12. Validation of TDP-43 inCITE analysis. (A) siTDP-43 knockdown in HBEC5i human brain EC line, compared to siControl treated HBEC5i cells, and analyzed in an inCITE-Seq experiment along with a standard brain nuclei preparation. (B) UMAP showing neuronal clusters analyzed, and violin plots showing the levels of nuclear TDP-43 protein (relative to nuclear H3) by disease state in these neuronal clusters. Violin plots include inner box plots that represent the interquartile range (25th–75th percentiles) with the median (50th percentile) as the center line. Whiskers represent 1.5xIQR with outliers shown.

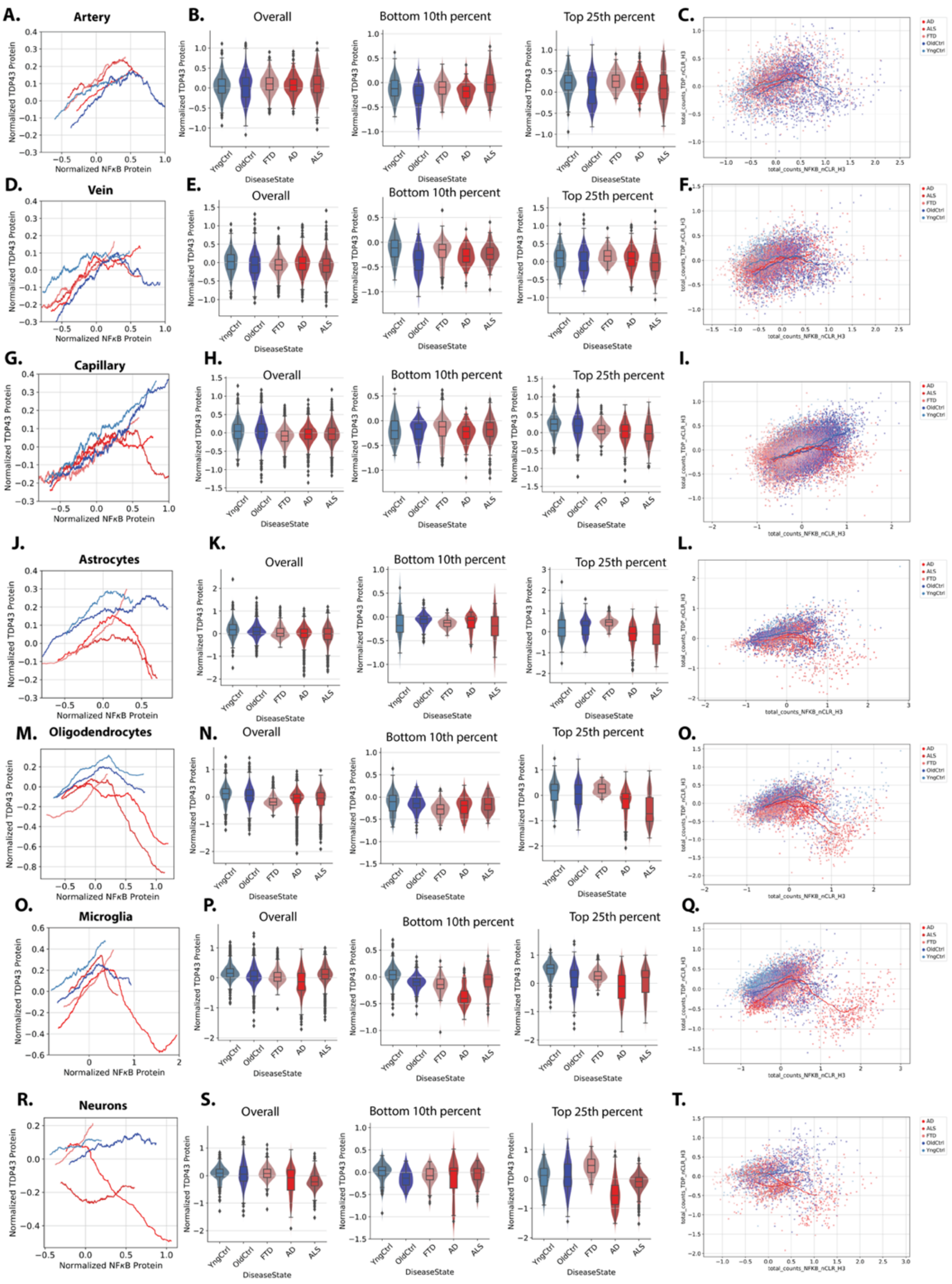

SI Figure 13. The relationship between TDP-43 and NF-kB using inCITE-Seq measurement in different cell types. Line plots display mean expression values across different disease conditions for Arteries (A), Veins (D), Capillaries (G), Astrocytes (J), Oligodendrocytes (M), Microglia (O), and Neurons (R). Violin plots highlight overall TDP-43 levels, the bottom 10th percentile, and the top 25th percentile of NF-κB, alongside raw data points for Arteries (B-C), Veins (E-F), Capillaries (H-I), Astrocytes (K-L), Oligodendrocytes (N-O), Microglia (P-Q), and Neurons (S-T). Violin plots include inner box plots that represent the interquartile range (25th–75th percentiles) with the median (50th percentile) as the center line. Whiskers represent 1.5xIQR with outliers shown.

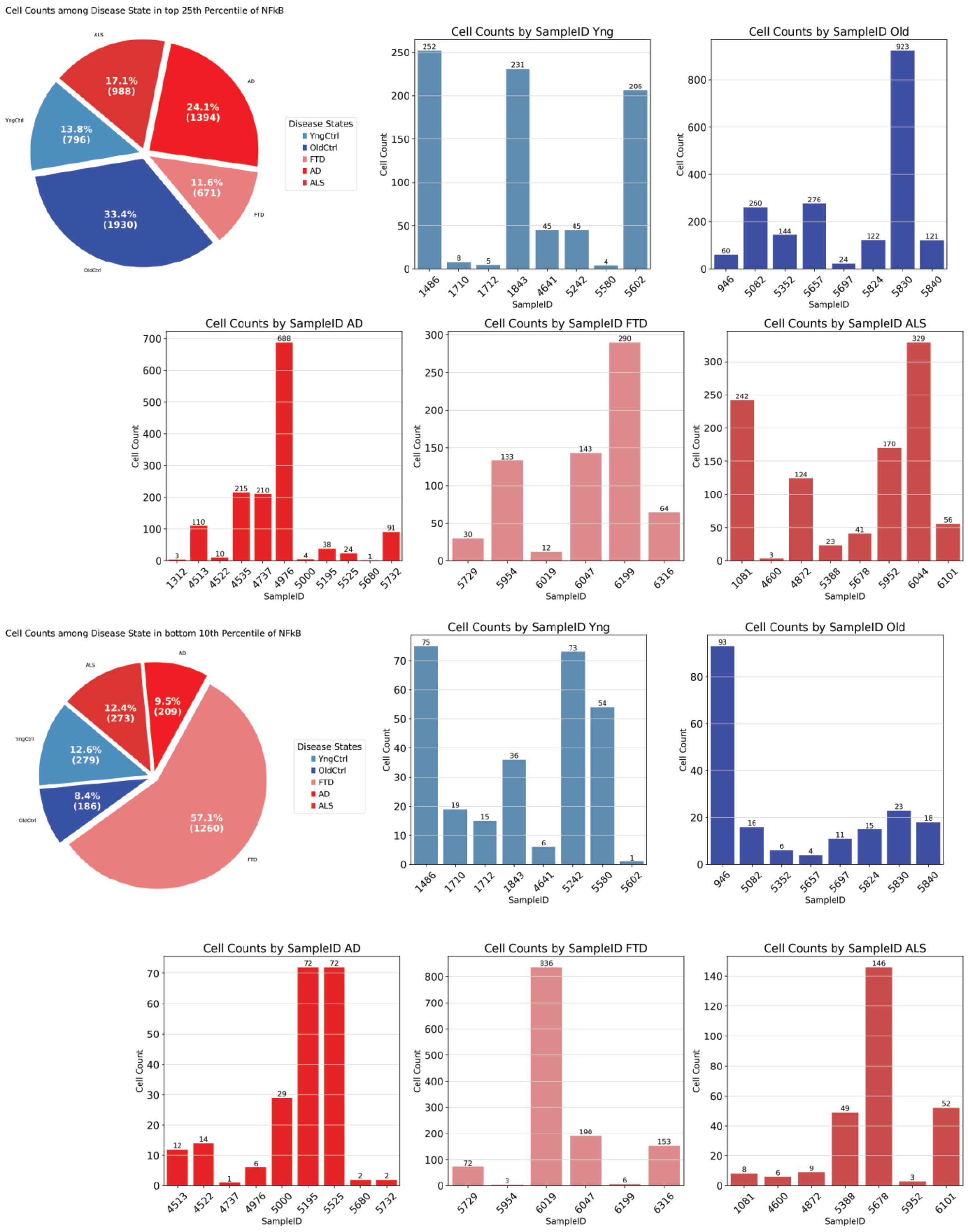

SI Figure 14. Composition of samples in extremes of p65/NF-kB. Pie charts and bar plots showing the proportion of cells in the top 25th percentile of p65/NF-kB:H3 and the bottom 10th percentile of p65/NF-kB:H3.

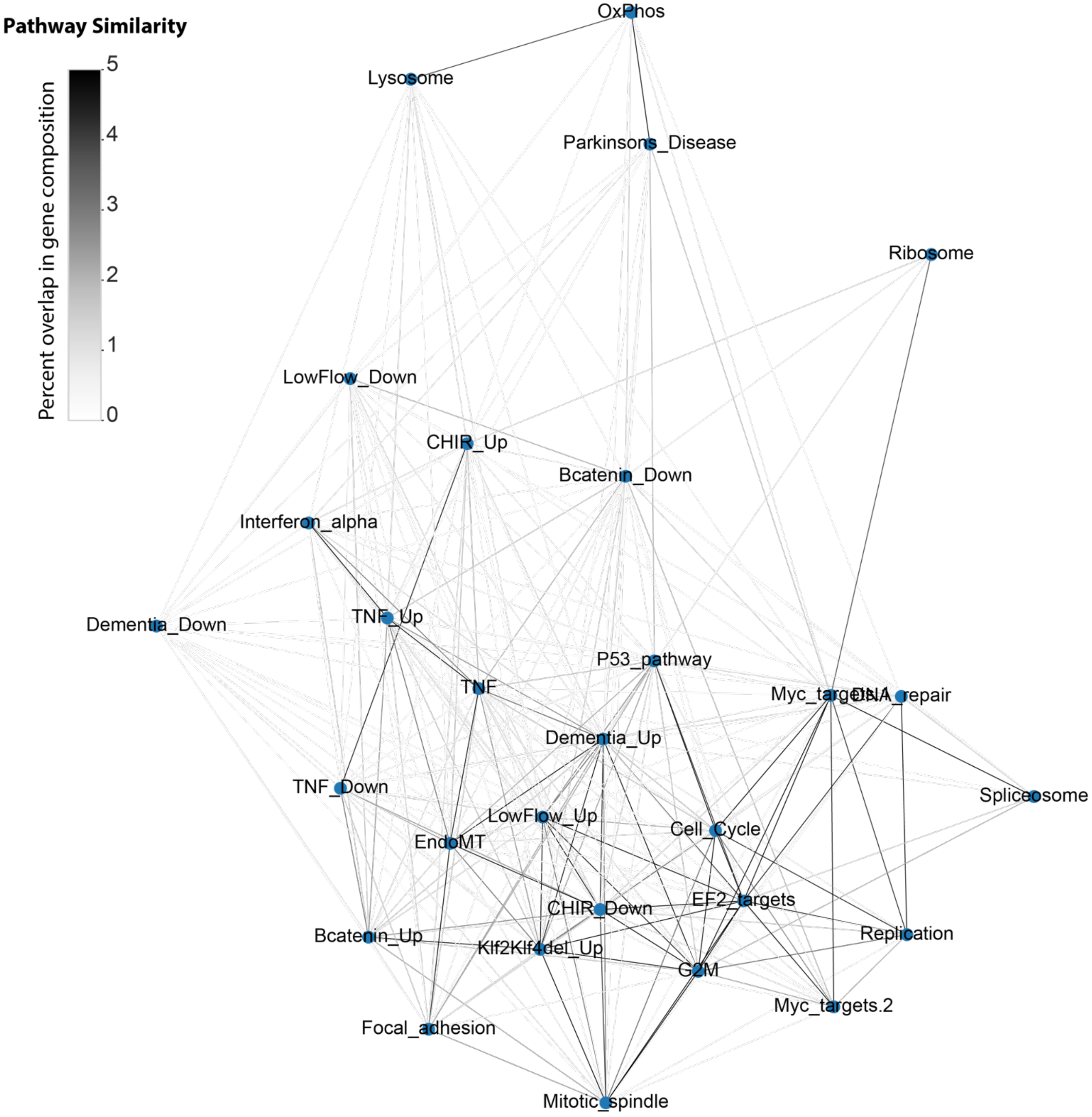

SI Figure 15. Overlap in GSEA terms. Plot, developed by NetworkX, shows the overlap between enriched GSEA terms in *in vitro* and *in vivo* models of TDP-43 disruption, and REV capillary ECs. The percent overlap in terms is indicated by the intensity of the lines (as a % of combined terms between any two nodes).

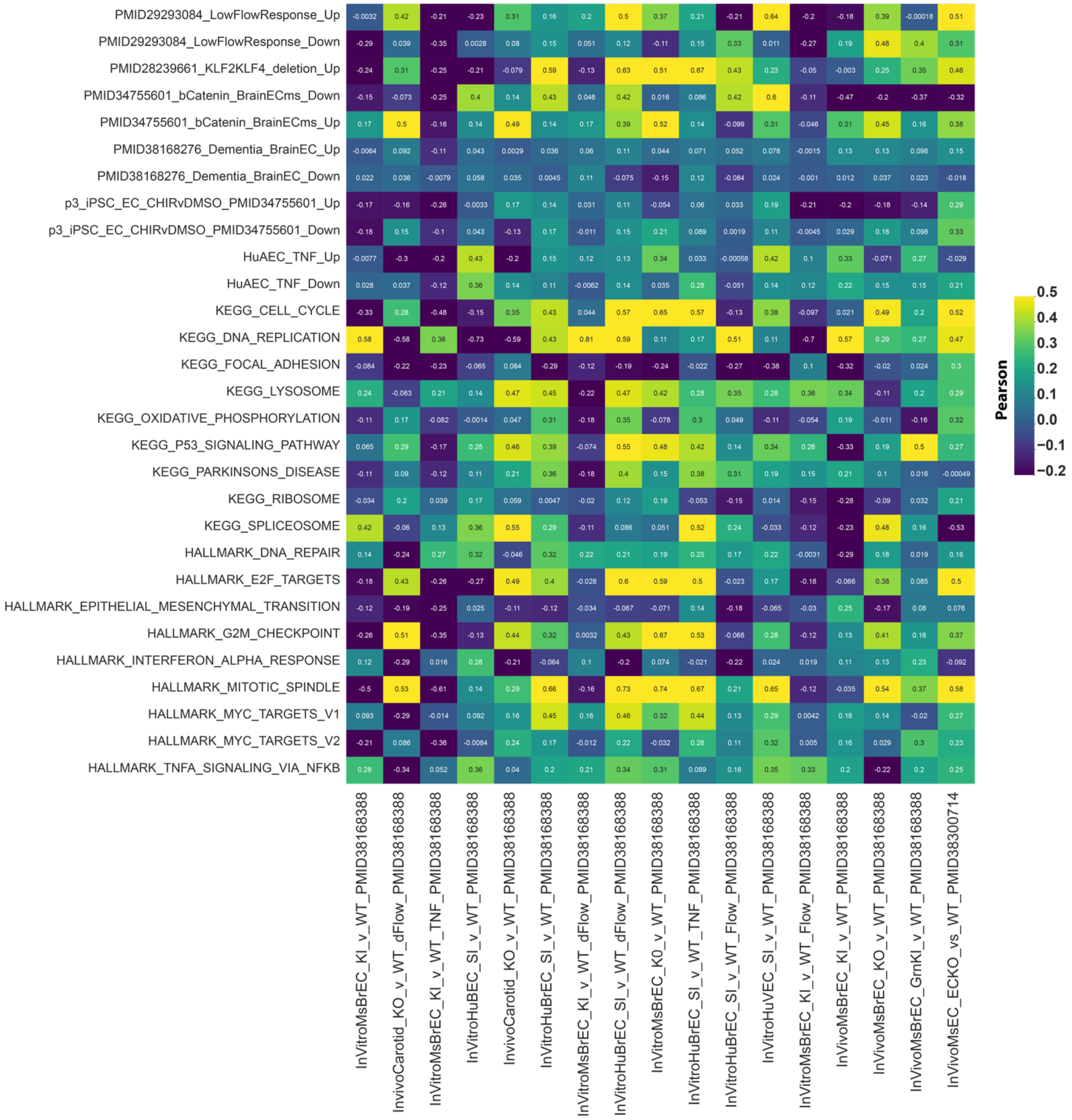

SI Figure 16. Correlation in response between TDP-43 disruption (KI, KO or siRNA) and REV capillary endothelial cells for select pathways. Heat map shows the correlation between gene expression in the REV capillary ECs (relative to Homeostatic ECs) and the indicated perturbations of TDP-43 (X-axis categories). Y-axis show the gene subset used to map each correlation. For example, for the 199 genes which characterize the low flow response (PMID29293084), the correlation between the log2-FC in human brain ECs with siTDP-43 and the log2-FC between REV and Homeostatic capillary ECs is R=0.5. The X-Y plot showing this data is in Figure 7H (for genes which mapped between mouse and human, using Biomart), but the correlation value for this, and the many other gene subsets is shown here.

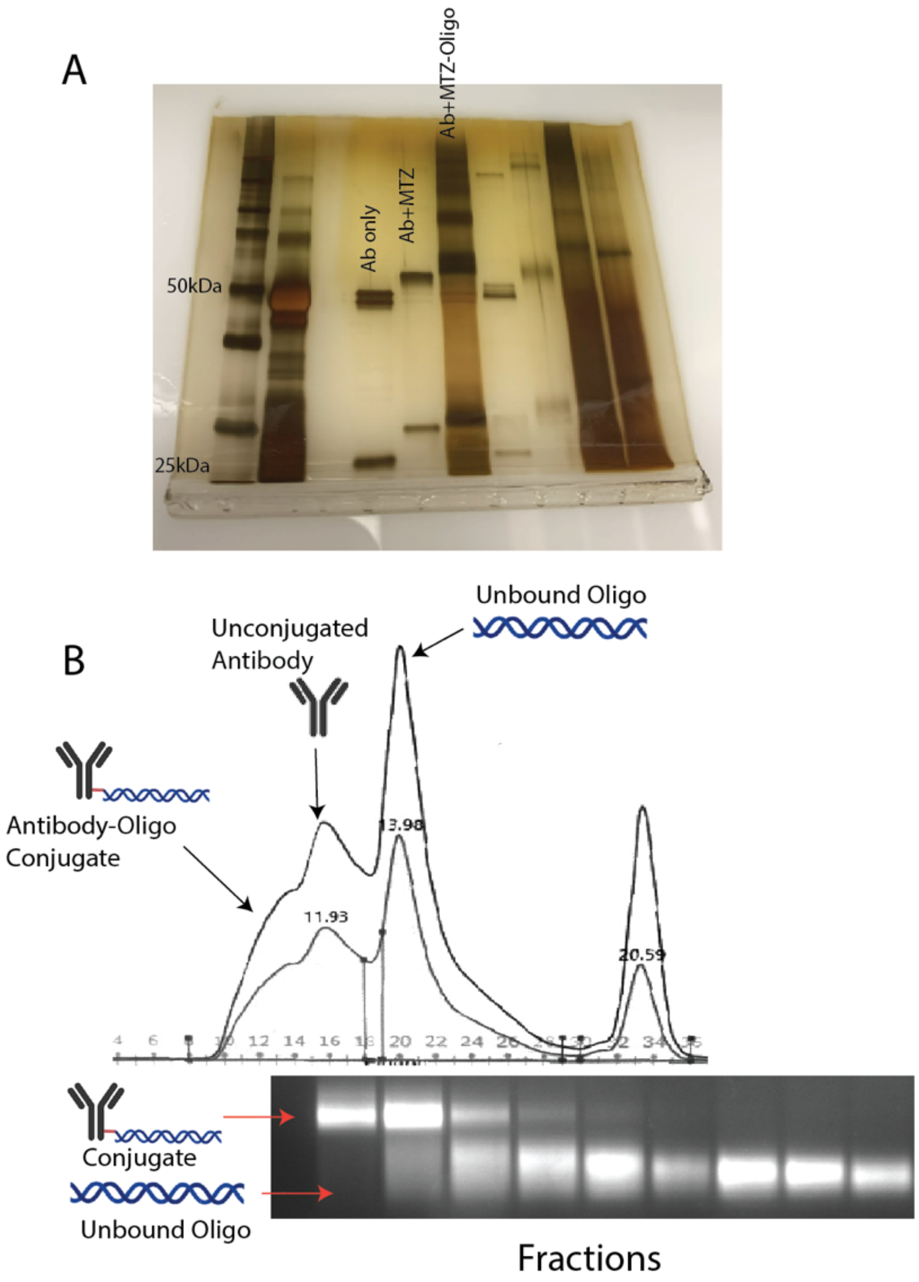

SI Figure 17. Size-exclusion purification of oligo-conjugated antibodies. (A) Reduced and silver-stained gel showing the step-wise addition of click chemistry adduct (MTZ) and oligo nucleotide (MTZ-Oligo). (B) Oligo-conjugated antibody cleanup via size exclusion chromatography. Top, a representative size exclusion chromatogram recorded with dual absorption wavelengths (260 and 280 nm). Conjugated antibodies, having the largest molecular weights, were eluted first from the column, followed by the free antibodies. Unconjugated oligonucleotides were the last to be eluted from the column. Bottom, fractions from the column were analyzed by an agarose gel stained with SybrGold showing the presence of free and conjugated oligonucleotides.

Supplementary Tables:

SI Table 1. Donor tissue attributes. Characteristics of Brodmann Area 10 samples from NIH NeuroBioBank at Maryland. Clinical diagnosis, age, sex, average gene and UMI counts per cell (n_gene and n_count), cell counts, RIN values (obtained from analysis of isolated RNA from samples and NIH assessment), post mortem interval, and analysis approach (3'expression or 3'expression+inCITE).

SI Table 2. inCITE-Seq antibodies. List of antibodies used for inCITE-seq analysis, including name, source, and barcodes.

SI Table 3. Pseudobulk analysis of AD donors compared to unaffected old donors by cell type. Sheets indicate the individual cell-type specific comparisons made.

SI Table 4. Pseudobulk analysis of ALS donors compared to unaffected old donors by cell type. Sheets indicate the individual cell-type specific comparisons made.

SI Table 5. Pseudobulk analysis of FTD donors compared to unaffected old donors by cell type. Sheets indicate the individual cell-type specific comparisons made.

SI Table 6. Pseudobulk analysis of young donors compared to unaffected old donors by cell type. Sheets indicate the individual cell-type specific comparisons made.

SI Table 7. Summary of RNA-sequencing data used for comparisons with TDP-43 deletion. Sheets indicate the samples and conditions for RNA-sequencing datasets, the per gene transcript counts obtained by STAR alignment and RSEM analysis (posterion mean count), and DESeq2 approach to identify differential expression.

SI Table 8. Source and composition of endothelial gene sets used for GSEA analyis. Columns indicate the source of the gene sets and their composition.
