# Supplementary material for: Endothelial TDP-43 depletion disrupts core blood–brain barrier pathways in neurodegeneration
Source: Nat Neurosci. 2025 Mar 14;28(5):973–84. doi: 10.1038/s41593-025-01914-5 (PMC12081287; doi:10.1038/s41593-025-01914-5)
Supplement: Supplementary file 2 — Reporting Summary [file 41593_2025_1914_MOESM2_ESM.pdf]

nature portfolio

Corresponding author(s): Patrick Murphy
Last updated by author(s): Oct 2, 2024

Reporting Summary

Nature Portfolio wishes to improve the reproducibility of the work that we publish. This form provides structure for consistency and transparency in reporting. For further information on Nature Portfolio policies, see our Editorial Policies and the Editorial Policy Checklist.

Statistics

For all statistical analyses, confirm that the following items are present in the figure legend, table legend, main text, or Methods section.

n/a	Confirmed	
☐	☒	The exact sample size (*n*) for each experimental group/condition, given as a discrete number and unit of measurement
☐	☒	A statement on whether measurements were taken from distinct samples or whether the same sample was measured repeatedly
☐	☒	The statistical test(s) used AND whether they are one- or two-sided<br>*Only common tests should be described solely by name; describe more complex techniques in the Methods section.*
☐	☒	A description of all covariates tested
☐	☒	A description of any assumptions or corrections, such as tests of normality and adjustment for multiple comparisons
☐	☒	A full description of the statistical parameters including central tendency (e.g. means) or other basic estimates (e.g. regression coefficient) AND variation (e.g. standard deviation) or associated estimates of uncertainty (e.g. confidence intervals)
☐	☒	For null hypothesis testing, the test statistic (e.g. *F*, *t*, *r*) with confidence intervals, effect sizes, degrees of freedom and *P* value noted<br>*Give P values as exact values whenever suitable.*
☒	☐	For Bayesian analysis, information on the choice of priors and Markov chain Monte Carlo settings
☐	☒	For hierarchical and complex designs, identification of the appropriate level for tests and full reporting of outcomes
☐	☒	Estimates of effect sizes (e.g. Cohen's *d*, Pearson's *r*), indicating how they were calculated

Our web collection on statistics for biologists contains articles on many of the points above.

Software and code

Policy information about availability of computer code

Data collection	Standard 10x single cell with dual index analysis pipeline was used, as described in the methods.<br><br>In this study, we used the following software tools: Cellranger, SciPy StatsModels, NumPy, Pandas, Scanpy, DemuxEM, Matplotlib, Seaborn, Scrublet ,Pegasus, and the commercial software Prism. In addition to published code from Chung et al 2021 (Nature Methods).
Data analysis	Data processing pipelines may be found on GitHib : https://github/pamurphyUCONN/2024_Omar<br><br>Cellranger for processing single-cell RNA sequencing data, Python libraries for statistics and data visualization: SciPy for executing scientific computing tasks, StatsModels for carrying out statistical modeling and tests, NumPy for conducting numerical operations, Pandas for handling data manipulation and analysis, Scanpy for the examination of single-cell genomics data, DemuxEM for the demultiplexing of barcoded sequencing data, Matplotlib and Seaborn for visualizing data, Scrublet for identifying sample doublets in single-cell RNA-seq data, and the commercial software Prism for other data visualization tasks.

For manuscripts utilizing custom algorithms or software that are central to the research but not yet described in published literature, software must be made available to editors and reviewers. We strongly encourage code deposition in a community repository (e.g. GitHub). See the Nature Portfolio guidelines for submitting code & software for further information.

Data

Policy information about availability of data

All manuscripts must include a data availability statement. This statement should provide the following information, where applicable:

- Accession codes, unique identifiers, or web links for publicly available datasets
- A description of any restrictions on data availability
- For clinical datasets or third party data, please ensure that the statement adheres to our policy

As described in Data Availability:

Data processing pipelines may be found on GitHib : https://github/pamurphyUCONN/2024_Omar
And also archived on Zenodo as https://doi.org/10.5281/zenodo.14679104

Source .h5 data files produced by Cellranger, after BCL2fastq and Cellranger count (as described in Materials and Methods) : https://murphylabvasculardata.cam.uchc.edu/download
And Zenodo as https://doi.org/10.5281/zenodo.14679170

Filtered and annotated h5ad files produced (as described in Materials and Methods) : https://murphylabvasculardata.cam.uchc.edu/download
And Zenodo as https://doi.org/10.5281/zenodo.14679170

Interactive data browser can be found at https://murphylabvasculardata.cam.uchc.edu

Raw fastq data and processed data files can be found in National Institute of Mental Health (NIMH) Data Archive under the Collection ID#5120 “Contributions of Endothelial RNA-binding Protein Dysregulation to Blood Brain Barrier Defects and Neurodegenerative Disease.”

Research involving human participants, their data, or biological material

Policy information about studies with human participants or human data. See also policy information about sex, gender (identity/presentation), and sexual orientation and race, ethnicity and racism.

Reporting on sex and gender	Sex, and not gender, is used in metadata, as obtained from the NIH NeuroBioBank, contained in Supplemental Table 1.
Reporting on race, ethnicity, or other socially relevant groupings	When available, data on race and ethnicity is reported in metadata, contained in the referenced h5ad file.
Population characteristics	Population characteristics are clearly described in metadata, which is contained in Supplemental Table 1.
Recruitment	NA - samples obtained are post-mortem from donors recruited by the NIH NeuroBioBank
Ethics oversight	NIH NeuroBioBank

Note that full information on the approval of the study protocol must also be provided in the manuscript.

Field-specific reporting

Please select the one below that is the best fit for your research. If you are not sure, read the appropriate sections before making your selection.

☒ Life sciences ☐ Behavioural & social sciences ☐ Ecological, evolutionary & environmental sciences

For a reference copy of the document with all sections, see nature.com/documents/nr-reporting-summary-flat.pdf

Life sciences study design

All studies must disclose on these points even when the disclosure is negative.

Sample size	Sample size was defined for an effect size of 1.5 and power of 80 for independent samples, using rank test. Total samples size was 92, with approximately 15 samples per groups.
Data exclusions	We excluded five samples for the following reasons: Sample '1103' was excluded due to concurrent conditions that could affect endothelial function, such as familial hypercholesterolemia. Sample '1233' was excluded because neuropathology reports indicated normal findings, inconsistent with the medical diagnosis of ALS. Sample '1946' was excluded as it was from a patient with Lewy body dementia. Samples '5077' and '4906' were excluded due to poor antibody counts.
Replication	Key findings for differential expression between cell populations were validated in two separate datasets. We also attempted to assess

Replication	findings in other publicly available single cell data sets from human cortex, but found that either the number of nuclei per donor were too low to be useful (<20 on average) or a low number of samples (N=4 pairs) with lower UMI counts than in our own data.
Randomization	To minimize batch effects, we aimed to include different disease groups and controls in each 10x Genomics sequencing reaction. Each individual was barcoded with a unique nuclear pore complex for downstream demultiplexing.
Blinding	NA

Reporting for specific materials, systems and methods

We require information from authors about some types of materials, experimental systems and methods used in many studies. Here, indicate whether each material, system or method listed is relevant to your study. If you are not sure if a list item applies to your research, read the appropriate section before selecting a response.

Materials & experimental systems

n/a	Involved in the study
☐	☒ Antibodies
☒	☐ Eukaryotic cell lines
☒	☐ Palaeontology and archaeology
☒	☐ Animals and other organisms
☒	☐ Clinical data
☒	☐ Dual use research of concern
☒	☐ Plants

Methods

n/a	Involved in the study
☒	☐ ChIP-seq
☐	☒ Flow cytometry
☒	☐ MRI-based neuroimaging

Antibodies

Antibodies used	All antibodies are described an an antibody table, Supplemental Table 2.
Validation	When possible, monoclonal antibodies chosen were KO validated (Abcam), or had previously be validated in publications. Key antibodies (TDP-43 and NFkB) were validated in data analysis of siRNA knockdown or well described cell phenotypes (e.g. NFkB high microglia in AD).

Plants

Seed stocks	*Report on the source of all seed stocks or other plant material used. If applicable, state the seed stock centre and catalogue number. If plant specimens were collected from the field, describe the collection location, date and sampling procedures.*
Novel plant genotypes	*Describe the methods by which all novel plant genotypes were produced. This includes those generated by transgenic approaches, gene editing, chemical/radiation-based mutagenesis and hybridization. For transgenic lines, describe the transformation method, the number of independent lines analyzed and the generation upon which experiments were performed. For gene-edited lines, describe the editor used, the endogenous sequence targeted for editing, the targeting guide RNA sequence (if applicable) and how the editor was applied.*
Authentication	*Describe any authentication procedures for each seed stock used or novel genotype generated. Describe any experiments used to assess the effect of a mutation and, where applicable, how potential secondary effects (e.g. second site T-DNA insertions, mosiacism, off-target gene editing) were examined.*

Flow Cytometry

Plots

Confirm that:

☒ The axis labels state the marker and fluorochrome used (e.g. CD4-FITC).

☒ The axis scales are clearly visible. Include numbers along axes only for bottom left plot of group (a 'group' is an analysis of identical markers).

☒ All plots are contour plots with outliers or pseudocolor plots.

☒ A numerical value for number of cells or percentage (with statistics) is provided.

Methodology

Sample preparation	Nuclei isolation generally followed our previously published protocol with minor modifications 17. All steps were performed either on ice or at a temperature of 4°C, unless specified otherwise. Frozen cortical brain tissues, weighing between 100 to 200 mg, were thawed at room temperature for 3-4 minutes in Nuclei EZ lysis buffer (Sigma, Nuc101) mixed with RNAase inhibitor (0.5U/µl, Clontech Cat #2313) in RINO tubes containing eight 3.2 mm stainless steel beads. The tissues were then

mechanically homogenized using a Next Advance Bullet Blender BB724M, set to level 4 for 4 minutes at 4°C. Following homogenization, the mixture was diluted in 5 mL, centrifuged at 500xG for 5 minutes (retaining the supernatant for analysis of total brain protein and mRNA), and subsequently washed with 5 mL of Nuclei EZ lysis buffer. A 2-minute incubation on ice preceded a second centrifugation at the same speed for another 5 minutes. The supernatant was then discarded, and the homogenate was resuspended in 5 mL of Nuclei EZ lysis buffer, followed by a 5-minute incubation on ice. The homogenate was then strained through a 70 µm pluristrainer, centrifuged at 4°C, and the supernatant removed. The resulting nuclei pellet was suspended in 200 µl of a PBS-based staining solution (1% BSA, PBS, 0.5U/µl RNAase inhibitor) in 1.5 ml low-bind tubes. After washing the nuclei with staining buffer (PBS + 0.2% BSA + RNAse inhibitor), the supernatant was removed, and 50 µl of blocking buffer, containing ssDNA (1 mg/mL final concentration) and FC block (1:100 of BioLegend, 156604), was added. This was followed by a 10-minute incubation. The nuclei were then stained with a buffer containing Anti-Erg 647 (1:200, Clone EPR3864, Abcam), Anti-NeuN Cy3 (1:200, Sigma), DAPI for nuclei labeling (1:2000 of a 5 mg/mL stock), and InCITE seq antibody mix. Before using the antibody mix, we incubated it with EcoSSB (Promega M3011) in 50µl of 1X NEBuffer 4 for 30 min at 37°C as suggested previously 18. Afterwards, 1 µl of TotalSeqB Hashtag (anti-Nuclear Pore Complex Proteins Hashtag at 0.5g, Biolegend 682239-682243) was added to each sample for subsequent identification in downstream analysis. After staining, the nuclei were washed twice with staining buffer and fixed with 2% PFA in staining buffer for 1 minute at 4C. Then, 500 µl of staining buffer containing 0.1% glycine was added to quench the fixation. The nuclei were centrifuged for 5 minutes at 500xG twice and resuspended in 500 µl of staining solution. This solution was then filtered through a 35 µm filter before sorting the nuclei using an Aria 2 with a 40 µm nozzle, collecting them into a BSA-coated tube maintained at 4°C.

Instrument

BD FACSAria 2 with a 40 µm nozzle.

Software

BD software and FlowJo

Cell population abundance

NA

Gating strategy

Gating was initially defined by separate staining of cells with and without Erg, and unstained controls. Clearly defined populations were sorted in subsequent experiments. We have previously published the gating strategy used, and reference this in the paper PMID 34775494.

☐ Tick this box to confirm that a figure exemplifying the gating strategy is provided in the Supplementary Information.
